# Assessing emerging and priority micropollutants in sewage sludge: environmental insights and analytical approaches

**DOI:** 10.1007/s11356-023-30963-1

**Published:** 2023-12-12

**Authors:** Diana Rede, Ivan Teixeira, Cristina Delerue-Matos, Virgínia Cruz Fernandes

**Affiliations:** 1 REQUIMTE/LAQV, Instituto Superior de Engenharia do Porto, Instituto Politécnico do Porto, Rua Dr. António Bernardino de Almeida, 431, 4249-015 Porto, Portugal; 2 Departamento de Química e Bioquímica, Faculdade de Ciências, Universidade do Porto, Rua do Campo Alegre S/N, 4169-007 Porto, Portugal

**Keywords:** PCBs, PAHs, Flame retardants, Pesticides, POPs, QuEChERS, Micropollutants, Sewage sludge

## Abstract

The application of sewage sludge (SS) in agriculture, as an alternative to manufactured fertilizers, is current practice worldwide. However, as wastewater is collected from households, industries, and hospitals, the resulting sludge could contaminate land with creeping levels of pharmaceuticals, pesticides, heavy metals, polycyclic aromatic hydrocarbons, and microplastics, among others. Thus, the sustainable management of SS requires the development of selective methods for the identification and quantification of pollutants, preventing ecological and/or health risks. This study presents a thorough evaluation of emerging and priority micropollutants in SS, through the lens of environmental insights, by developing and implementing an integrated analytical approach. A quick, easy, cheap, effective, rugged, and safe (QuEChERS) extraction method, coupled with gas chromatography and liquid chromatography, was optimized for the determination of 42 organic compounds. These include organophosphorus pesticides, organochlorine pesticides, pyrethroid pesticides, organophosphate ester flame retardants, polybrominated diphenyl ethers, polychlorinated biphenyls, and polycyclic aromatic hydrocarbons. The optimization of the dispersive-solid phase for clean-up, combined with the optimization of chromatographic parameters, ensured improved sensitivity. Method validation included assessments for recovery, reproducibility, limit of detection (LOD), and limit of quantification (LOQ). Recoveries ranged from 59.5 to 117%, while LODs ranged from 0.00700 to 0.271 µg g^-1^. Application of the method to seven SS samples from Portuguese wastewater treatment plants revealed the presence of sixteen compounds, including persistent organic pollutants. The quantification of α-endosulfan, an organochlorine pesticide, was consistently observed in all samples, with concentrations ranging from 0.110 to 0.571 µg g^-1^. Furthermore, the study encompasses the analysis of agronomic parameters, as well as the mineral and metal content in SS samples. The study demonstrates that the levels of heavy metals comply with legal limits. By conducting a comprehensive investigation into the presence of micropollutants in SS, this study contributes to a deeper understanding of the environmental and sustainable implications associated with SS management.

**Graphical Abstract:**

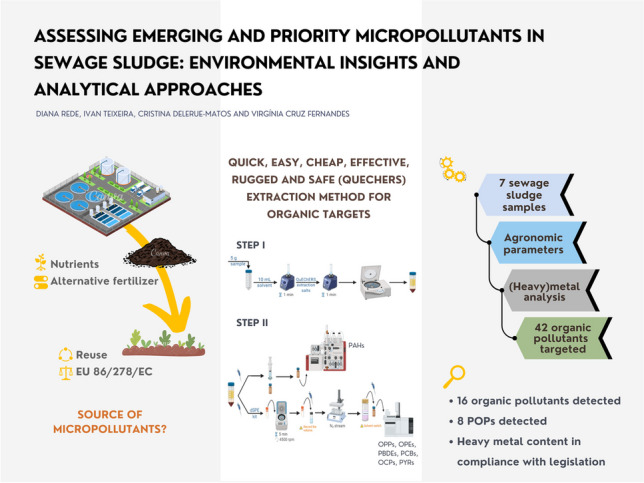

**Supplementary Information:**

The online version contains supplementary material available at 10.1007/s11356-023-30963-1.

## Introduction

Sewage sludge (SS) is a solid, semi-solid, or slurry by-product that emerges from treatment processes conducted in wastewater treatment plants (WWTPs) (Martín-Pozo et al. 2019; Campo et al. 2021). In a conventional WWTP, the influent undergoes the following steps: (i) pretreatment (removal of large debris and grit), (ii) primary treatment (separation of suspended solid matter in sedimentation tanks), (iii) secondary treatment (biological cleaning through an activated sludge system), and (iv) (bio)solids handling (thickening, stabilization, dewatering, and disposal) (Venkatesan and Halden 2020; Campo et al. 2021). SS is produced during primary and secondary treatments, and its exact composition varies and is not known. The wastewater collected can come from households, industries, healthcare facilities, rainwater runoff, or landfill leachate (Venkatesan and Halden 2020; Hatinoğlu and Sanin 2021). In addition, any chemical compounds used in everyday products such as pharmaceuticals, pesticides, heavy metals, polycyclic aromatic hydrocarbons (PAHs), and microplastics that are not eliminated during wastewater treatment processes can sorb SS (Rossini et al. 2016; Horton et al. 2017; van den Berg et al. 2020; Venkatesan and Halden 2020). Commonly, the disposal routes for SS are land application (such as agricultural, forestry, and reclamation), energy recovery (including incineration and co-combustion), and landfilling. However, improper dumping is still practiced in developing regions (Martín-Pozo et al. 2019; Zhu et al. 2019; Schnell et al. 2020).

Millions of tons of SS are produced annually. For example, the European Union (EU) produces approximately 9 million tons of dry matter of SS. These quantities are expected to increase in the short term (Mohapatra et al. 2016; Chen et al. 2021). According to the United Nations, three out of ten people do not have access to safe drinking water services, and six out of ten people lack access to sanitation facilities. It is estimated that more than 80% of the wastewater resulting from human activities is discharged into the aquatic environment without undergoing any prior treatment (UN DESA 2023). Moreover, existing WWTPs are unable to effectively remove emerging contaminants at trace levels (Paíga et al. 2019). By 2030, with a growing global population, there is an anticipated shortage of approximately 40% of freshwater resources (UN DESA 2023). To meet the strict regulations imposed by the governments and to ensure universal access to water and sanitation (fulfilling the sixth goal of the 2030 Agenda for Sustainable Development), it will be crucial to construct new WWTPs and enhance the existing ones. This will increase SS production (Wyrwicka et al. 2014; Gago et al. 2019). However, the challenge in managing and disposing of SS lies in effectively handling the large quantities generated while adhering to the principles of the circular economy, which prioritize strategies such as reuse and recycling (Campo et al. 2021).

SS has high organic matter and nutrient content; hence, it is considered a valuable resource rather than a waste and is widely used as a fertilizer to enhance crop, pasture, and rangeland production (Martín-Pozo et al. 2019; Hatinoğlu and Sanin 2021). In the EU, the reuse of SS on agricultural land is highly encouraged, in line with the principles of the circular economy. However, to safeguard humans, animals, plants, and the environment, the spread of SS must comply with Council regulations, specifically the Sewage Sludge Directive 86/278/EEC and the EU Working Document on Sewage Sludge. These regulations establish limit values for agronomic parameters, heavy metals, and organic compounds, including polychlorinated dibenzodioxins and dibenzofurans (European Commission 1986; European Comission 2000). Even so, studies have highlighted that potentially toxic elements (PTEs) sorbed onto SS can be transferred to soil, causing its deterioration and the subsequent loss of function (Mahon et al. 2017; Martín-Pozo et al. 2019; Corradini et al. 2019). This can ultimately result in ecological and/or health risks, such as the spread of antibiotic resistance genes (Buta et al. 2021; Yuan et al. 2023), the prevalence of SARS-CoV-2 or its genetic material (Adelodun et al. 2022), the bioaccumulation of metals and PAHs on crops and biota (Zhang et al. 2017; Iglesias et al. 2018; Courtois et al. 2021), or the absorption of microplastics by fungi (Wan et al. 2022). Moreover, it must not be forgotten that hundreds of new synthetic compounds are launched in the global market every year. For example, the USA alone is responsible for the production of 1500 new products annually (Naidu et al. 2021).

To minimize the environmental impact of incorporating SS into soils, it is essential to develop efficient analytical methodologies that enable the extraction and analysis of various contaminants. Typically, the techniques used to extract organic compounds from SS are solid-phase extraction (Liang and Liu 2016), matrix solid-phase dispersion (Sánchez-Brunete et al. 2009), solid–liquid extraction (Chen et al. 2019), the Soxhlet method, and ultrasonic-assisted extraction. The Quick, Easy, Cheap, Effective, Rugged, and Safe (QuEChERS) method, which was initially designed for extracting and purifying contaminants from food samples, has also been successfully used for environmental samples (Guillemet et al. 2009; Guo et al. 2009; Suciu et al. 2015; Barakat et al. 2017; Tomczyk et al. 2020). This method, known for its speed, reliability, and cost-effectiveness, as it does not rely on expensive instrumentation, has previously been applied to sludge samples for the determination of pharmaceuticals, ultraviolet filters, and synthetic musks (Rossini et al. 2016; Ramos et al. 2019). The detection of emerging pollutants in SS is generally achieved using gas chromatography (GC) (Chen et al. 2019) and high-performance liquid chromatography (HPLC) (Castro et al. 2021), typically in conjunction with mass spectrometry (MS). The QuEChERS extraction technique demonstrates excellent performance in terms of efficiency, versatility, and reduction of interferents (such as fats or pigments) that may be present in the sample matrix. This technique produces an extract that is suitable for chromatographic analysis.

Since the environment, especially SS is facing an increasing number of toxic micropollutants that pose proven or potential risks, this study has two main objectives. Firstly, we have developed a simple and rapid method that combines the QuEChERS extraction technique with chromatographic analysis to identify 42 organic compounds from seven chemical families in SS. These include six organophosphorus pesticides (OPPs), five organochlorine pesticides (OCPs), five pyrethroid pesticides (PYRs), seven organophosphate esters (OPEs), seven polybrominated diphenyl ethers (PBDEs), four polychlorinated biphenyls (PCBs), and eight polycyclic aromatic hydrocarbons (PAHs) in SS. Additionally, we assess the content of agronomic parameters (pH in water (pH_w_), dry matter (DM), organic matter (OM) and total organic carbon (TOC)), and mineral and metal content (Co, Mo, Hg, Cd, Ni, Pb, Cr, Cu, Zn, Li, Be, V, As, Se, Rb, Sr, Rh, Sb, Cs, Ba, Ir, Tl, Mg, Ca, Mn, and Fe) using standardized methods. Secondly, our study contributes with data to future considerations on legislation on SS and its application on agricultural land, in the framework of the Action Plan for a New Circular Economy adopted on March 11, 2020, to protect the environment and human health. The developed method was applied to analyze samples from seven different Portuguese WWTPs, collectively serving over 380,000 inhabitants. To the best of our knowledge, our study represents a pioneering effort in two key areas. Firstly, we employed the QuEChERS method for the simultaneous extraction of these emerging and priority organic compounds from SS samples. Secondly, we conducted a comprehensive analysis of Portuguese SS for the presence of multiple organic micropollutants not currently addressed in the existing legislation. Using geographically representative samples from a specific region in Portugal, our findings contribute to a better understanding of contamination and have the potential to drive future legislative measures aimed at reducing these emerging pollutants in SS, providing crucial data for the development of targeted mitigation measures in the region.

## Materials and methods

### Reagents and materials

Deionized water with a resistivity of 15.8 MΩ cm was obtained using an Elix® 3ADV system (Millipore SAS, Molsheim, France) equipped with a Q-Gard® T1 purification cartridge and a Vent Filter MPK01 for the storage tank (Merck Millipore, Darmstadt, Germany). Nitric acid (HNO_3_) (65% w/v) was supplied by Merck (Darmstadt, Germany). Acetonitrile (ACN) from VWR Chemicals, Leicestershire, England, and n-hexane from Merck, Darmstadt, Germany, were of chromatography grade. Reference materials BCR®–667 (sediment) and ISE 918 (soil) were obtained from the Institute for Reference Materials and Measurements (Belgium) and WEPAL-QUASIMEME (Netherlands), respectively. The QuEChERS EN Method salts, primary secondary amine (PSA), C18, SampliQ anhydrous magnesium sulphate (MgSO_4_), and SampliQ Carbon SPE Bulk Sorbent (CB) were purchased from Agilent Technologies (Santa Clara, California, USA). Additionally, Supel™ QuE Z-Sep (Z-Sep) was acquired from Supelco (Bellefonte, Pennsylvania, USA). OPPs (dimethoate, chlorpyrifos-methyl, parathion-methyl, malathion, chlorpyrifos, and chlorfenvinphos), OPEs (tri-iso-butyl phosphate (TiBP), tri-n-butyl phosphate (TnBP), tris(2-chloroethyl) phosphate (TCEP), triphenyl phosphate (TPhP), tris(2-butoxyethyl) phosphate (TBEP), tris(2-ethylhexyl) phosphate (TEHP), and tri-cresyl phosphate (TCP)), PBDEs ((2,4,4′-tribromodiphenyl ether (BDE 28), 2,2′,4,4′-tetrabromodiphenyl ether (BDE 47), 2,2′,4,4′,5-pentabromodiphenyl ether (BDE 99), 2,2′,4,4′,6-pentabromodiphenyl ether (BDE100), 2,2′,4,4′,5,5′-hexabromodiphenyl ether (BDE 153), 2,2′,4,′,5,6′-hexabromodiphenyl ether (BDE 154), 2,2′,3,4,4′,5′,6-heptabromodiphenyl ether (BDE 183)), PCBs (2,4,4′-trichlorobiphenyl (PCB 28), 2,3′,4,4′,5′-pentachlorobiphenyl (PCB 118), 2,2′,4,4′,5,5′-hexachlorobiphenyl (PCB 153), and 3,4,4′,5,5′-heptachlorobiphenyl (PCB 180)), OCPs (hexachlorocyclohexanes (HCH), *α*-endosulfan, 2,2-bis(p-chlorophenyl)-1,1-dichloroethylene (p,p′'-DDE), dieldrin, and dichlorodiphenyldichloro-ethane (p,p′-DDD)), PYRs (bifenthrin, *λ*-cyhalothrin, cypermethrin, fenvalerate, and deltamethrin) standards, and dichlorobenzophone (internal standard (IS)) were acquired from Sigma Aldrich and Chemservice (West Chester, Pennsylvania, USA). PAHs (fluoranthene (FLU), benzo[b]fluoranthene, benzo[j]fluoranthene, benzo(a)pyrene (BaP), dibenzo[a,l]pyrene, dibenz[a,h]anthracene (DBA), benzo[ghi]perylene, indeno[1,2,3‐cd]pyrene) were obtained from Supelco (Bellefonte, Pennsylvania, USA). All standards were of analytical grade.

### Sampling and characteristics of sewage sludges

Sewage sludge samples, which were properly stabilized and dewatered, were collected from seven WWTPs located in three different regions of Northern Portugal. Five of these WWTPs can be found in the Porto region, while the other two are situated in the Viseu and Aveiro regions, as illustrated in Fig. 1S in the Supporting Information. However, all of them receive urban wastewater as well as some industrial and agricultural discharges. The samples were identified from A to G and stored in pre-cleaned glass containers at - 20 °C until analysis. Sample A was collected from a WWTP that serves a population of approximately 285,000 inhabitants. The plant receives an average flow of 39,470 m^3^ per day and operates with secondary treatment using the activated sludge process. Samples B, C, D, E, and G were collected from WWTPs with secondary treatment based on an extended aeration system. These plants receive average flows between 380 and 8270 m^3^ per day and serve populations of approximately 54,660, 3430, 2260, 2890, and 3430 inhabitants, respectively. Sample F was collected from a WWTP that serves a population of approximately 35,430 inhabitants (3750 m^3^ day^-1^). The WWTP operates with a secondary treatment system, which consists of a trickling filter process followed by an activated sludge process. A mixture of samples, referred to as the *sludge mixture*, was prepared to develop a method for quantifying organic pollutants. The moisture percentages of the individual SS samples were used to calculate adjusted quantities for the sludge mixture, ensuring consistent contributions based on dry-weight (dw). The Supporting Information contains details in section S.1.2 about the methods used to determine pH_w_ values, DM, OM, TOC contents, and concentrations of trace elements in SS samples.

**Fig. 1:**
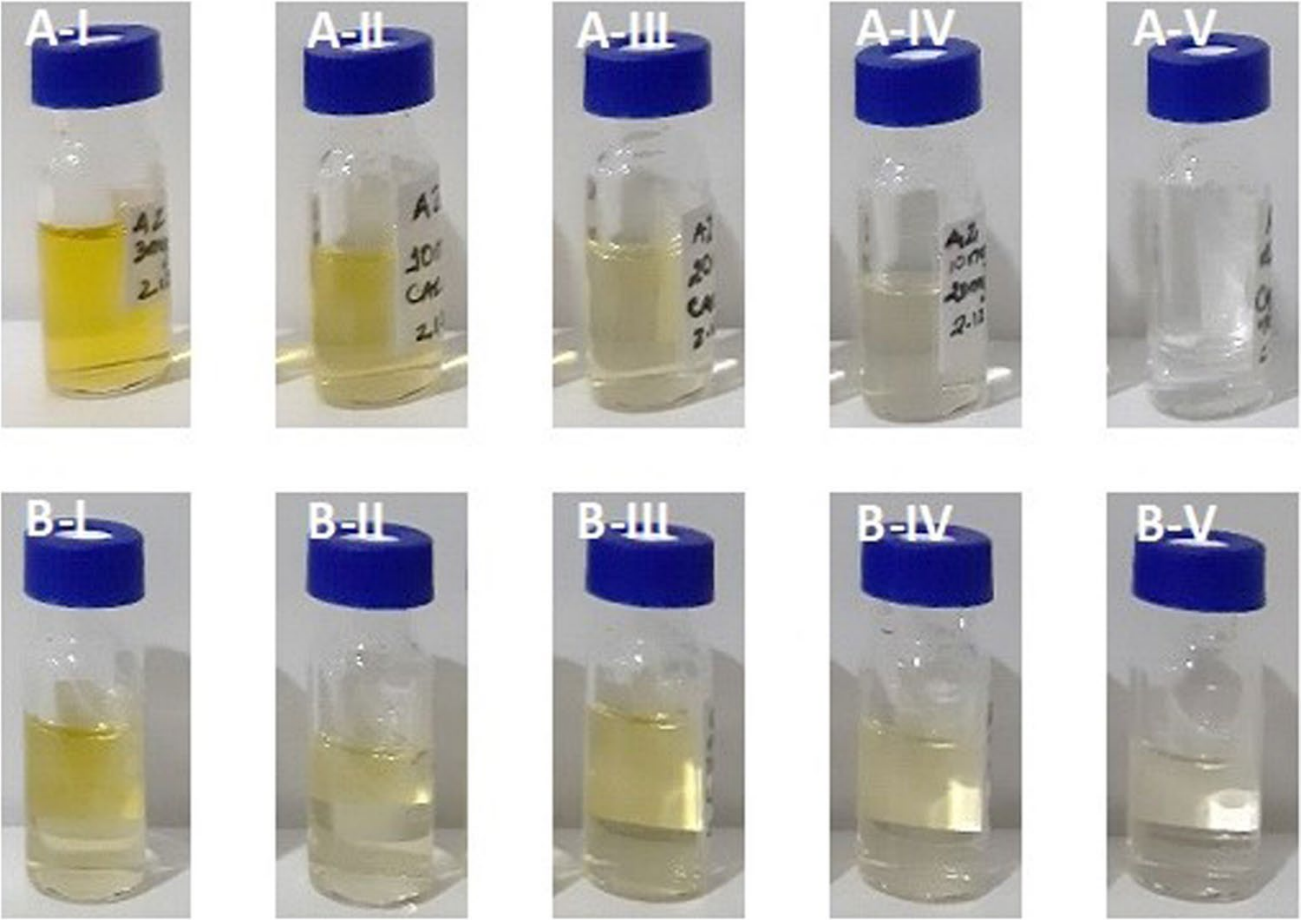
Color removal with different d-SPE mixtures applied to samples A and B in the first set of clean-ups. The d-SPE tube contained a typical mixture of 150 mg of MgSO_4_, 50 mg of PSA, and 50 mg of C18 with A-I — 3 mg of CB; A-II — 10 mg of CB; A-III — 20 mg of CB; A-IV — 20 mg of CB and 10 mg of Z-Sep; A-V — 50 mg of CB; B-I — 3 mg of CB; B-II — 6 mg of CB; B-III — 10 mg of CB; B-IV — 10 mg of CB and 10 mg of Z-Sep; B-V — 50 mg of CB

### Standard solutions for the extraction and detection of organic pollutants

Individual stock solutions (2500 µg L^-1^) of each OPPs, OPEs, PBDEs, PCBs, and OCPs were prepared in n-hexane, while PYRs were prepared in ACN. Reagent-only and matrix-matched calibration standards were prepared for OPPs and OPEs, with concentrations ranging from 20 to 100 µg L^-1^ (0.207–1.034 µg g^-1^). For PBDEs, PCBs, OCPs, and PYRs, the calibration standards ranged from 10 to 100 µg L^-1^ (0.103–1.034 µg g^-1^). Calibration standards for PAHs were prepared with concentrations ranging from 0.13 to 20.06 µg L^-1^ (0.00026–0.040 µg g^-1^, values for BaP). To prepare matrix-matched calibration standards, aliquots of the stock solutions were dried using a nitrogen stream and then re-dissolved in the *sludge mixture* extract.

### Sample preparation for the extraction and detection of organic pollutants

The extraction methodology was based on the QuEChERS method described by Fernandes et al. (2012). Briefly, 5 g of the SS sample (wet weight) were placed in a glass tube. Next, 10 mL of ACN was added to the sample and vortexed for 1 min. QuEChERS EN salts (4 g of MgSO_4_, 1 g of NaCl, 1 g of trisodium citrate dihydrate, and 0.5 g of disodium hydrogen citrate sesquihydrate) were added. The tube was then vortexed for 1 min. The mixture was centrifuged at 2000 rpm for 15 min.

For the assessment of PAHs, 1 mL of supernatant was filtered using a 0.22-μm BGB Analytik PTFE syringe filter with a 13-mm diameter and collected in a 1.5-mL glass vial. The final extract was injected into a liquid chromatograph equipped with photodiode array (PAD) and fluorescence (FLD) detectors, referred to as LC-PDA-FLD (Oliveira et al. 2015). For the analysis of OPPs, OPEs, PBDEs, PCBs, OCPs, and PYRs analysis, the extracts underwent a cleaning process to minimize matrix interference. This was achieved through dispersive solid-phase extraction (d-SPE) using a mixture of sorbents in bulk form. The composition of this mixture was optimized, as shown in Table 1. Initially, a series of five different mixtures (I-V) were tested in SS, A and B. The performance of the procedure was evaluated based on its color removal capability, which determined the selection of sorbents to be included in the d-SPE. Then, in the second set of four bulk mixtures (VI-IX), the recovery rates (RE) and matrix effects (ME) of each target analyte were evaluated within the sludge mixture by adjusting the amounts of sorbents in the d-SPE. The supernatant (1 mL) was added to a 4-mL glass vial containing a mixture of bulk sorbents. It was then vortexed for 1 min and centrifuged at 4500 rpm for 5 min. The clear supernatant was transferred to a 1.5-mL amber glass vial, evaporated to dryness under a gentle nitrogen stream, redissolved with n-hexane, and vortexed. Approximately 150 μL of extract was transferred to a second amber glass vial containing a 250-μL insert and injected into the following instruments for analysis: (i) a gas chromatograph (GC-2010) with a flame photometric detector (FPD) from Shimadzu to quantify OPPs and OPEs; (ii) a gas chromatograph (GC-2010) with an electron capture detector (ECD) from Shimadzu for the quantification of PBDEs, PCBs, OCPs, and PYRs (the internal standard was added prior to analysis); (iii) a gas chromatograph with a mass spectrometry detector (GC-MS) from Thermo Fisher to confirm chlorpyrifos-methyl, malathion, TiBP, TPhP, TEHP, BDE 28, BDE 99, BDE 183, α-endosulfan, PCB 118, cypermethrin, and deltamethrin in the samples. The Supporting Information provides details about the chromatographic methods used in section S.1.3.

**Table 1:** Composition of the d-SPE mixtures tested in the two sets

First set					
d-SPE mixture	MgSO_4_ PSA C18 (mg)	SS A		SS B	
		CB (mg)	Z-Sep (mg)	CB (mg)	Z-Sep (mg)
I	150 50 50	3	0	3	0
II		10	0	6	0
III		20	0	10	0
IV		20	10	10	10
V		50	0	50	0
Second set (*sludge mixture*)					
d-SPE mixture	MgSO_4_ (mg)	PSA (mg)	C18 (mg)	CB (mg)	Z-Sep (mg)
VI	150	50	50	10	10
VII	150	50	50	5	15
VIII	150	50	50	5	20
IX	150	50	50	5	25

### Validation of analytical methods for the extraction and detection of organic pollutants

The method was validated according to European Union guidelines (Magnusson and Örnemark 2014; European Commission 2021) for linearity and range, matrix effects (ME), trueness, precision, limit of detection (LOD), and quantification (LOQ) (Bhardwaj et al. 2016; European Commission 2019). Eight concentration levels of matrix-matched standards and reagent-only standards were used to obtain calibration curves, which plotted the peak area against the concentration of the target analyte. The calibration curves enabled the establishment of linearity, expressed as the coefficient of determination (*R*^2^), within the selected range of concentrations. ME was investigated by comparing the slopes between calibration in the matrix and calibration in the reagent for each target analyte (Ponce-Robles et al. 2017).

The trueness of the analytical procedure, expressed as RE, was evaluated by conducting recovery studies. To conduct these studies, the *sludge mixture* was fortified both before (*pre-spike*) and after (*post-spike*) the analytical procedure. Fortifications were made at two levels: OPPs and OPEs at 50 and 75 µg L^-1^ (0.517 and 0.776 µg g^-1^); OCPs, PBDEs, PCBs, and PYRs at 20 and 50 µg L^-1^ (0.207 and 0.517 µg g^-1^); and PAHs at 0.50 and 2.51 µg L^-1^ (0.0010 and 0.0050 µg g^-1^, values for BaP). Two replicates were conducted for each level on two different days. The RE was determined by comparing the peak areas before and after the spike. In addition, the sorbent mixture used in the d-SPE clean-up was selected based on the ME obtained by comparing the peak areas of the post-spike and the standard in the solvent at a specific concentration level. Precision was studied in terms of intraday and day-to-day repeatability, which are expressed as the relative standard deviation (% RSD) of a series of measurements.

For the analyses performed in GC-FPD, GC-ECD, and LC-PDA-FLD, the LOD and LOQ were determined by considering the residual standard deviation of the linear regression and the slope of the calibration curve (Miller and Miller 2010; Magnusson and Örnemark 2014). Both LOD and LOQ were expressed as the concentration of the target analyte in the sample. Additionally, TCP, deltamethrin, fenvalerate, and λ-cyhalothrin were quantified by summing the peak areas of their respective isomers. Positive results were confirmed by GC-MS, and the identification was carried out using selected ion monitoring (SIM), which involved the use of three ions that were sufficiently selective for each analyte (European Commission 2021).

## Results and discussion

### Optimization of analytical methodologies for the extraction and detection of organic pollutants

The chromatographic methods for GC-FPD (OPPs and OPEs) and GC-ECD (PBDEs, PCBs, OCPs, and PYRs) were based on previous studies conducted by the research group (Fernandes et al. 2012, 2018, 2020; Correia-Sá et al. 2012; Bragança et al. 2019; Cruz Fernandes et al. 2020 et al. 2020; Dorosh et al. 2021; Sousa et al. 2023). However, due to the high complexity of the SS matrix, modifications were made to achieve maximum sensitivity for identifying and quantifying multiple target analytes. The organic fraction of SS depends on the source of the effluent. However, it typically consists of proteins, humic substances, carbohydrates, and significant amounts of cellular lipids, free fatty acids, and wax/gums. This results in a highly intricate and diverse matrix (Zhu et al. 2017; Chen et al. 2021; Gonzalez et al. 2021).

The sample preparation method was performed using the QuEChERS approach, which is a salting-out extraction technique with an organic solvent (first stage) combined with d-SPE clean-up using sorbents (second stage). In this study, QuEChERS EN salts (4 g MgSO_4_, 1 g NaCl, 1 g trisodium citrate dihydrate, 0.5 g of disodium hydrogen citrate sesquihydrate) were selected based on previous works described in the literature (Herrero et al. 2014; Maragou et al. 2021). The clean-up step, which is crucial for reducing the matrix effect when analyzing complex samples (Herrero et al. 2014; Ponce-Robles et al. 2017), was optimized in terms of the sorbents and their quantities. The experiments were performed using a standard mixture of 150 mg of MgSO_4_, 50 mg of PSA, and 50 mg of C18, combined with varying amounts of CB (used for pigment removal) and Z-Sep (used for lipid content removal) (Ponce-Robles et al. 2017; Fernandes et al. 2020). In the first set of d-SPE mixtures tested, it was observed that increasing the amount of CB in the mixture led to a reduction in the intensity of the sample color (Fig. 1). In fact, 50 mg of CB in the d-SPE bulk mixture (A-V and B-V) achieved almost complete color removal. However, it should be noted that using high amounts of CB can lead to the adsorption of the target analytes (Masiá et al. 2015). Thus, since the combinations of CB/Z-Sep also decrease the color intensity in both sample extracts (A-IV: 20 mg/10 mg; B-IV: 10 mg/10 mg), we tested different amounts of these sorbents in the second set of d-SPE. Recovery rates and ME were assessed for each cleanup procedure using a fortified *sludge mixture* before and after the QuEChERS method. The concentration used for the fortified mixture was 50 μg L^-1^ (0.517 µg g^-1^) for OPPs and OPEs, and 20 µg L^-1^ (0.207 µg g^-1^) for PBDEs, PCBs, OCPs, and PYRs. Matching reagent-only standards were prepared.

**Fig. 2:**
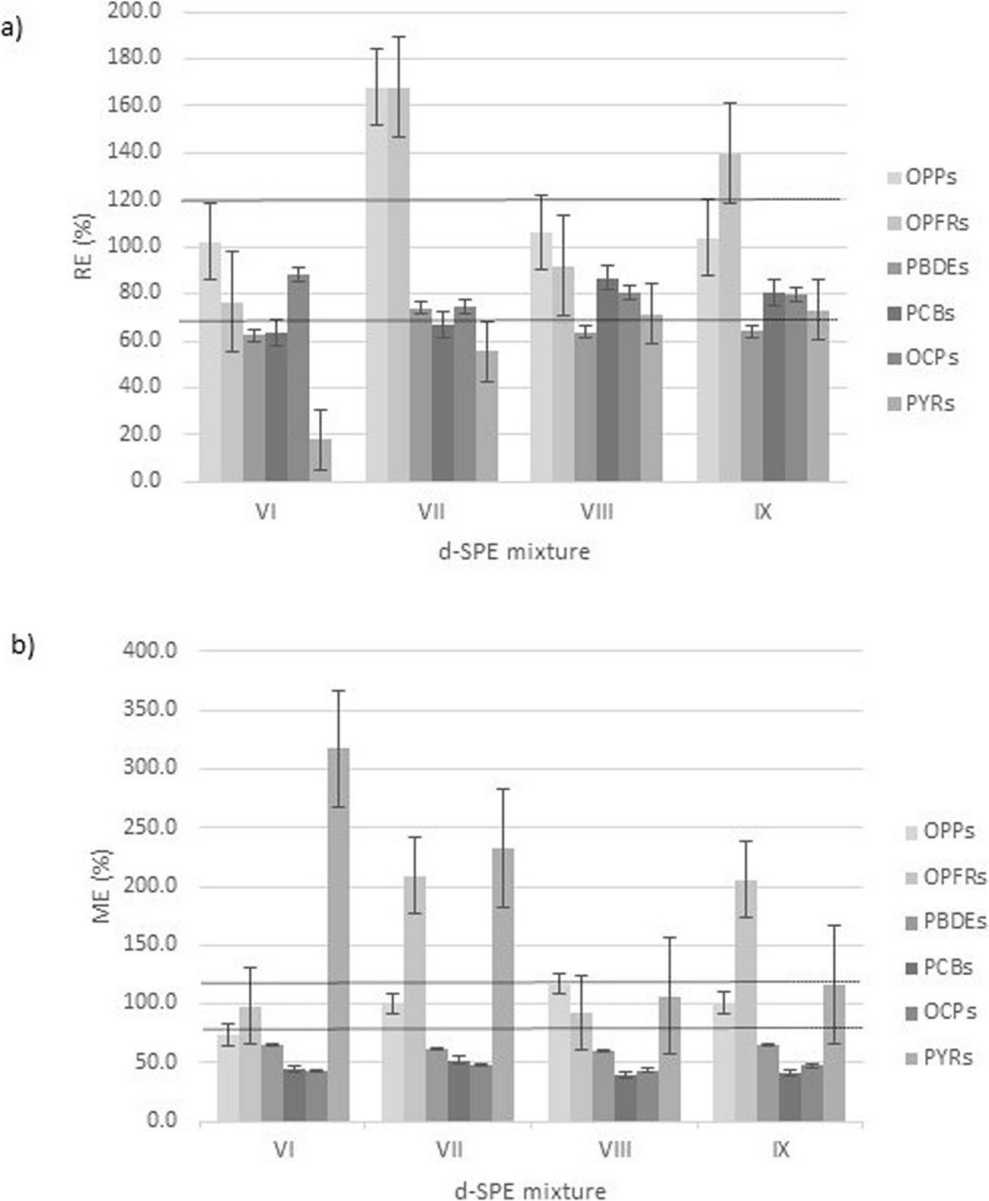
RE (%) (**a**) and ME (**b**) obtained with the different formulations of d-SPE sorbents (VI–IX) used in the clean-up of the *sludge mixture*. The d-SPE tube contained a typical mixture of 150 mg of MgSO_4_, 50 mg of PSA, and 50 mg of C18 combined with VI – 10 mg of CB and 10 mg of Z-Sep; VII – 5 mg of CB and 15 mg of Z-Sep; VIII– 5 mg of CB and 20 mg of Z-Sep; IX – 5 mg of CB and 25 mg of Z-Sep

Figure 2a shows the recovery results for different mixtures of bulk sorbents, with mean recoveries ranging from 18.1 to 102.1% (VI), 55.6 to 372.3% (VII), 71.3 to 106.3% (VIII), and 63.9 to 139.7% (IX). Overall, mixtures with lower CB content and higher Z-Sep showed improved RE (%), particularly for PYRs (18.1% (VI) < 55.6% (VII) < 71.3% (VIII) < 73.1% (IX)). Mixture VIII exhibited the lowest RSDs with values of 11.6% for OPPs, 14.7% for OPEs, 13.8% for PBDEs, 26.9% for PCBs, 14.4% for OCPs and 8.0% for PYRs. Figure 2b shows the results of the ME determinations, where chromatographic interferences were observed in all d-SPE mixtures. However, these interferences were reduced in mixture VIII, as no effect was observed for OPPs, OPEs, and PYRs (117.3%, 92.6%, and 107% respectively). On the other hand, signal suppression was noticed for PBDEs, PCBs, and OCPs (60.3%, 39.3%, and 43.8% respectively). Mixture VIII was selected for the clean-up step because it provided acceptable recovery values and had less pronounced matrix interference. This is the first study using a sorbent mixture consisting of 150 mg of MgSO_4_, 50 mg of PSA, 50 mg of C18, 5 mg of CB, and 20 mg of Z-Sep for extracting OPPs, OPEs, PBDEs, PCBs, OCPs, and PYRs from SS samples.

### Validation of the analytical methodologies for the extraction and detection of organic pollutants

The development of a quantification method requires a validation process that ensures precision, robustness, and minimization of differences in the matrix effect and/or extraction efficiency among samples (Herrero et al. 2014). The developed method was validated for each compound in terms of the linear range, ME, LODs, LOQs, RE, repeatability (intraday), and reproducibility (day-to-day) (Magnusson and Örnemark 2014; Bhardwaj et al. 2016; European Commission 2019; European Commission 2021). All validation parameters are listed in Table 2.

**Table 2:** Coefficient of determination (*R*^2^), limit of detection (LOD) and quantification (LOQ), recovery (RE), matrix effect (ME), repeatability, and reproducibility (%RSD, *n* = 2) of the developed method for the studied compounds

	Target analyte	*R*^2^	LOD (μg g^-1^)	LOQ (μg g^-1^)	RE (%)	ME (%)	Repeatability (%RSD)	Reproducibility (%RSD)
OPPs	Dimethoate	0.9919	0.101	0.307	80.1	164.3**	7.80	18.2
	Chlorpyrifos methyl	0.9915	0.136	0.413	90.6	142**	3.40	6.70
	Parathion methyl	0.9634	0.271	0.821	86.0	193**	0.100	10.4
	Malathion	0.9946	0.109	0.331	87.1	155**	0.500	17.0
	Chlorpyrifos	0.9905	0.145	0.439	107	120	2.40	9.10
	Chlorfenvinphos	0.9947	0.123	0.373	115	115	11.0	14.4
OPEs	TiBP	0.9667	0.102	0.730	91.3	104	7.60	21.1
	TnBP	0.9950	0.106	0.322	86.3	133**	8.80	12.5
	TCEP	0.9925	0.130	0.394	74.0	152**	9.40	10.5
	TPhP	0.9958	0.096	0.292	79.8	107	2.50	5.80
	TBEP	0.9935	0.130	0.393	71.3	192**	6.40	11.6
	TEHP	0.9970	0.0770	0.257	61.9	145**	9.40	6.50
	TCP	0.9942	0.112	0.339	79.9	99.3	8.80	7.30
PCBs	PCB 28^1^	0.9940	0.102	0.341	126	91.91	4.61	11.0
	PCB 118^1^	0.9997	0.0220	0.0750	84.7	49.8*	2.53	12.7
	PCB 153^1^	0.9988	0.0440	0.145	68.0	67.2*	6.36	4.54
	PCB 180^1^	0.9999	0.0150	0.0500	57.5	55.3*	2.31	4.02
PBDEs	BDE 28	0.9998	0.0200	0.0670	72.9	65.6*	5.92	6.78
	BDE 47	0.9999	0.0100	0.0330	62.8	73.1*	6.69	6.56
	BDE 100	0.9973	0.0650	0.216	60.4	48.6*	1.49	3.87
	BDE 99	0.9999	0.0150	0.0500	62.0	63.4*	5.71	7.83
	BDE 154	0.9996	0.0260	0.0850	54.1	66.0*	2.26	8.23
	BDE 153	0.9993	0.0340	0.112	54.6	72.3*	0.120	7.54
	BDE 183	1.0000	0.00800	0.0260	49.7	61.1	3.02	20.0
OCPs	o-HCH	0.9958	0.0800	0.266	86.3	51.5*	3.65	5.59
	α-Endosulfan	0.9997	0.0220	0.074	81.6	56.3*	3.56	14.0
	p,p´-DDE	0.9991	0.0460	0.153	72.1	44.7*	4.13	5.19
	p,p´-DDD	0.9992	0.0350	0.118	67.1	52.4*	2.50	9.0
	Dieldrin	0.9973	0.0640	0.213	73.7	64.9*	5.65	6.0
PYRs	Bifenthrin	0.9949	0.0770	0.255	74.5	97.9	9.02	16.4
	λ-Cyhalothrin	0.9992	0.0420	0.140	63.4	72.1*	8.92	8.16
	Cypermethrin	0.9997	0.0210	0.0710	69.6	126.1**	3.81	10.9
	Fenvalerate	0.9988	0.0420	0.139	64.7	93.7	1.00	6.86
	Deltamethrin	0.9999	0.0150	0.0510	68.0	102.1	4.44	14.8
PAHs	Fluoranthene^1^	0.9816	0.0870	0.290	49.5	75.5*	2.00	7.30
	Benzo[b + j]fluoranthene^1^	0.9982	0.0590	0.196	94.8	139**	2.60	10.6
	Benzo[a]pyrene^1^	0.9985	0.00700	0.0240	103.1	101	1.00	1.20
	Dibenzo[a,l]pyrene	0.9997	0.0110	0.0380	80.0	96.2	4.60	2.40
	Dibenz[a,h]anthracene	0.9988	0.0230	0.0760	118.5	95.1	7.40	18.6
	Benzo[ghi]perylene^1^	0.9994	0.0170	0.0570	96.5	101	4.10	9.90
	Indeno[1,2,3‐cd]pyrene^1^	0.9972	0.0140	0.0470	91.6	101	6.40	11.9

^1^Included in working document on sludge

^*^Signal suppression

^**^Signal enhancement

Linearity was assessed using matrix-matched calibration solutions at eight concentration levels ranging from 0.207 to 1.034 µg g^-1^ for OPPs and OPEs; 0.103 to 1.034 µg g^-1^ for PBDEs, PCBs, OCPs, and PYRs; and 0.00026 to 0.040 µg g^-1^ (values for BaP) for PAHs. The use of matrix-matched calibration reduces the problems related to signal suppression or enhancement (Rajski et al. 2013; European Commission 2019). It has been described as a suitable strategy for analyzing complex matrices, such as SS (Herrero et al. 2013, 2014; Masiá et al. 2015). The coefficients of determination (*R*^2^) were higher than 0.9905 for all compounds, except for parathion methyl, TiBP, and FLU, which had *R*^2^ values of 0.9634, 0.9667, and 0.9816, respectively. The effects of the SS matrix on the extraction efficiency were evaluated by comparing the ratio of the slopes of the matrix-matched and reagent-only calibration curves. An ME closer to 100% suggests no influence from the matrix; an ME within the range of 80–120% indicates a soft matrix influence, while an ME outside this range reveals medium or strong influence, such as suppression or reinforcement (Rajski et al. 2013; Masiá et al. 2015). Thus, based on the obtained results, the following conclusions can be drawn: five compounds (TCP, deltamethrin, BaP, benzo[ghi]perylene, and indeno[1,2,3‐cd]pyrene) did not exhibit ME; ten compounds (TiBP, TPhP, chlorpyrifos, chlorfenvinphos, bifenthrin, fenvalerate, dibenzo[a,l]pyrene, and DBA) showed soft ME; seventeen compounds showed suppressed responses ranging from - 25% (FLU) to - 55% (p,p’-DDE), with an average value of - 38.8%; and ten compounds displayed enhanced responses ranging from + 26% (cypermethrin) to + 93.2% (parathion methyl), with an average value of + 54.1%.

The accuracy was assessed by conducting recovery tests at two levels of contamination. The recoveries were determined by comparing the concentration obtained from the spiked *sludge mixture* (before the extraction process) with the concentration obtained from the spiked extract. The samples were quantified using a combined matrix-matched calibration. According to these guidelines, the mean recoveries of target analytes within the scope of a method should be in the range of 70–120% (RSD ≤ 20%) (European Commission 2019). The recoveries obtained for approximately 40% of the target analytes were outside this range, varying from 49.6% (FLU) to 126% (PCB 28). This variation may be due to the diversity of chemical families included in the study and is consistent with the results reported for the analysis of multiple pharmaceutical families in SS (Peysson and Vulliet 2013). For each chemical family, the average recovery obtained was as follows: PAHs (90.6 ± 21.6%), OPPs (94.3 ± 13.6%), PCBs (84.0 ± 30.0%), OPEs (78.3 ± 8.6%), OCPs (76.8 ± 8.7%), PYRs (69.0 ± 4.6%), and PBDEs (59.5 ± 7.6%).

The precision of the chromatographic methods, expressed as % RSD, was determined by repeatedly injecting a spiked extract of *sludge mixture* at a single concentration level. The concentration levels were 0.207 µg g^-1^ for OPPs and OPEs; 0.103 µg g^-1^ for PBDEs, PCBs, OCPs, and PYRs; and 0.005 µg g^-1^ for PAHs (value for BaP). The obtained intraday RSD values were lower than 20% for all compounds, as were the inter-day RSD values, except for TiBP, which was 21.1%. The obtained results are acceptable, considering the complexity of the matrix. Therefore, the method can be considered precise. The LODs and LOQs of the GC-FPD, GC-ECD, and LCPDA–FLD methods were determined as 3.3 and 10 times the standard deviation of the residual (*S*_*y/x*_) divided by the slope of the linear regression (*b*), respectively. The LODs ranged from 0.007 to 0.271 µg g^-1^, while the LOQs ranged from 0.024 to 0.821 µg g^-1^, depending on the specific compound.

To the best of our knowledge, this is the first instance in which the QuEChERS approach, using citrate buffer and a d-SPE mixture of PSA, C18, MgSO_4_, CB, and Z-Sep, has been utilized for the simultaneous extraction of six OPPs, four OCPs, six PYRs, seven OPEs, seven PBDEs, four PCBs, and eight PAHs in SS. Nevertheless, there are studies in the literature that support the applicability of the QuEChERS approach for extracting organic pollutants from SS samples. Masiá et al. (2015) used QuEChERS to identify 50 pesticides, five of which were targeted in our study (dimethoate, parathion-methyl, malathion, chlorpyrifos, and chlorfenvinphos). They used 150 mg of MgSO_4_, 50 mg of C18, and 50 mg of PSA for cleanup and obtained RE ranging from 36 to 120% with 26% inter-day precision. For the five pesticides we both studied, their RE ranged from 40 to 101%. This addition of CB to the d-SPE mixture improves the % RE of OPPs. Ramos et al. (2019) used the QuEChERS method to extract synthetic musk compounds from SS. The d-SPE contained 500 mg of MgSO_4_, 410 mg of C18, and 315 mg of PSA, and the recoveries ranged from 75 to 122%, with interday and intraday precision of 1–8%. Peysson and Vulliet (2013) applied the QuEChERS approach to determine 17 hormonal steroids and 119 pharmaceutical compounds in SS, obtaining recoveries ranging from 15 to 131%. Herrero et al. (2014) evaluated the QuEChERS method, which involved using citrate buffer for extraction and Z-sep + as a cleaning material, to detect benzotriazoles, benzothiazoles, and benzenesulfonamide derivatives in SS. They achieved over 80% RE for all compounds, with less than 15% intra- and inter-day RSD values. The results of our study indicate that the proposed methodology is a suitable and flexible approach for quantifying OPPs, OPEs, PBDEs, PCBs, OCPs, PYRs, and PAHs in SS, with RE comparable or better than those reported in the literature. The applicability of the method was demonstrated by analyzing seven samples and comparing the results with published data.

### Assessment of physical, chemical, and micropollutant levels in sewage sludge samples

#### Analysis of physical, chemical, and elemental characteristics of sewage sludge samples

The physicochemical analysis of SS samples collected from different WWTPs included pH_w_, DM, OM, TOC, nutrients, heavy metals, and other trace elements, such as (Table S2 in the Supporting Information). The variation in sample composition may be related to the origin of the wastewater and the type of treatment process used. For instance, the heavy metal content mainly depends on the proximity of WWTPs to industrial sites (Halecki et al. 2016). The mean pH_w_ values varied between 6.08 (B and E) and 8.42 (A), indicating a slightly acidic to slightly alkaline pH_w_. The DM content of the SS samples ranged from 13.4 to 24.1%, and the OM content varied in the range of 68.7 to 85.8%. Sample A, collected from a WWTP that utilizes an activated sludge process for secondary treatment, exhibited the highest DM content and the lowest OM content. In Portugal, the agricultural use of SS from WWTPs is regulated by Decree-Law No. 276/2009. This law transposes Council Directive 86/278/EEC into national legislation (Ministério do Ambiente do Ordenamento do Território e do Desenvolvimento Regional 2009). According to the current legislation, the concentration of heavy metals must not exceed the following limits: 16 mg kg^-1^ dw for Hg, 20 mg kg^-1^ dw for Cd, 300 mg kg^-1^ dw for Ni, 750 mg kg^-1^ dw for Pb, 1000 mg kg^-1^ dw for Cr and Cu, and 2500 mg kg^-1^ dw for Zn. In the current study, the heavy metal content was found to be within the legal limits, significantly lower than the maximum allowed by the regulations, and consistent with the values reported in the literature (Halecki et al. 2016). Overall, the sample with the highest concentration of PTEs is C followed by A, G, D, F, E, and B.

Generally, studies on the effect of SS application on soil have focused on the variation in topsoil properties (~ 0–20 cm), once it corresponds to the incorporation depth of organic materials, but what about subsoil properties? Hechmi et al. (2021) studied unvegetated semi-arid soils treated with excessive doses of SS for four years. OM, N, P, and K accumulation was monitored in two soil profiles (0–20 and 20–40 cm), while Cu, Zn, Pb, and Ni contents and enzyme activities were assessed. Results showed that the SS application improved OM content in light-textured soils, enriching surface, and subsoil profiles with macronutrients. Moreover, the accumulation of heavy metals from SS did not inhibit essential microbial activities. This suggests that reusing SS can improve soil quality in semi-arid regions without causing significant environmental impacts. Additionally, Zhao et al. (2021) investigated the effect of SS on bacterial communities in forest plantation soils. Results showed an increased abundance of Bacteroidetes, Actinobacteria, and Chloroflexi, but not Acidobacteria, Proteobacteria, and Verrucomicrobia. Changes in keystone bacterial groups and an increase in the interactions among community members were observed, indicating community instability and adaptation to new conditions. These findings are of particular importance as microorganisms play a crucial role in soil fertility and stability. They are involved in biogeochemical cycles and the degradation of contaminants (Courtois et al. 2021). What about the effects on plants? Iglesias et al. (2018) analyzed 13 PTEs in barley and maize crops and soils amended with SS for 15 years. The results showed an increase in the amount of Pb, Hg, Zn, and Ag in cropland soils. However, the total PTEs contents remained below the thresholds established by US and European regulations. Yet, these regulations do not consider the mobility, extractability, and transfer of PTEs to plants, as pointed out by the authors.

#### Levels of organic pollutants in sewage sludge samples

Table 3 displays the levels of organic pollutants that were detected and quantified in SS samples after being adjusted for recoveries. Within this monitoring, 16 target compounds were detected: two OPPs (chlorpyrifos-methyl and malathion), four OPEs (TiBP, TPhP, TBEP, and TEHP), one PCB (28), three PBDEs (28, 99, and 183), one OCP (α-endosulfan), two PYRs (cypermethrin and deltamethrin), and three PAHs (FLU, BaP, and DBA). Among the compounds that were detected, eight were identified as persistent organic pollutants (POPs). In each sample, at least two target compounds were detected. Sample C presented the highest number of target compounds and the highest concentration of POPs, whereas F showed the highest concentration of pollutants, followed by E, C, B, A, G, and D. The differences between the samples may be attributed to the composition of the wastewater and/or the characteristics of each treatment system. The presence of pollutants from OPPs, OPEs, PCBs, PBDEs, and PYRs was confirmed through GC-MS analysis. Only TBEP has not been confirmed. To the best of our knowledge, no previous studies have been published on the concentrations of the target compounds in SS from WWTPs located in other regions of Portugal. The EU Sludge Directive establishes maximum concentration values for various substances in sludge intended for agricultural reuse. These substances include halogenated organic compounds, linear alkylbenzene sulfonate, di(2-ethylhexyl)phthalate, nonylphenols and nonyl ethoxylates with one or two ethoxy groups, PAHs (acenaphthene, phenanthrene, fluorene, FLU, pyrene, benzo(b + j + k)fluoranthene, BaP, benzo(ghi)perylene, and indeno(1, 2, 3-c, d)pyrene); PCBs (congeners 28, 52, 101, 118, 138, 153, and 180), and polychlorinated dibenzodioxins and dibenzofurans. Compounds belonging to the OPPs, OPEs, PYRs, OCPs, and PBDEs chemical families are not covered by the Sludge Directive. Table S3 in the Supporting Information displays the results for the 42 compounds, and Table S4 shows concentrations from the literature.

**Table 3:** Concentrations (μg g^-1^ dw) of OPPs, OPEs, PBDEs, PCBs, OCPs, PYRs, and PAHS in sewage sludge collected from municipal wastewater treatment plants located in the north of Portugal (*n* = 6; ND– not detected; < LOQ– below method quantification limit)

Target analyte		SS samples						
		A	B	C	D	E	F	G
OPPs	Chlorpyrifos methyl	ND	ND	< LOQ	ND	ND	ND	ND
	Malathion	ND	ND	ND	< LOQ	ND	ND	ND
OPEs	TiBP	ND	ND	ND	ND	ND	26.8 ± 0.013	ND
	TPhP	ND	ND	ND	< LOQ	2.05 ± 0.012	ND	ND
	TBEP	ND	1.06 ± 0.19	ND	ND	ND	ND	ND
	TEHP	ND	ND	ND	ND	ND	1.53 ± 0.023	ND
PCBs	PCB 118	0.302 ± 0.056	ND	ND	ND	ND	ND	ND
PBDEs	BDE 28	0.128 ± 0.027	< LOQ	ND	ND	ND	ND	ND
	BDE 99	ND	ND	0.0540 ± 0.012	ND	ND	< LOQ	ND
	BDE 183	< LOQ	ND	ND	ND	ND	ND	ND
OCPs	α-Endosulfan	0.110 ± 0.007	0.170 ± 0.005	0.490 ± 0.084	0.571 ± 0.037	0.366 ± 0.010	0.282 ± 0.019	0.286 ± 0.022
PYRs	Cypermethrin	0.0870 ± 0.012	ND	0.0573 ± 0.011	ND	ND	< LOQ	ND
	Deltamethrin	0.254 ± 0.0560	ND	ND	< LOQ	ND	ND	ND
PAHs	Fluoranthene	ND	ND	0.435 ± 0.0042	ND	ND	ND	0.401 ± 0.0143
	Benzo[a]pyrene	ND	ND	0.0442 ± 0.0002	ND	0.0418 ± 0.00003	ND	ND
	Dibenz[a,h]anthracene	ND	ND	< LOQ	ND	ND	ND	< LOQ
Total pollutant load per SS sample (μg g^-1^ dw)		0.881	1.26	2.25	0.571	2.46	28.6	0.687
Total POP load per SS sample (μg g^-1^ dw)		0.540	0.170	1.02	0.571	0.408	0.282	0.687
Number of pollutants detected per SS sample		6	3	7	4	3	5	2

##### Organophosphorus pesticides

Dimethoate, parathion-methyl, chlorpyrifos, and chlorfenvinphos were not detected in any sample. However, chlorpyrifos-methyl and malathion were detected in samples C and D, respectively, but their quantities were not determined. OPPs are used to control insect pests, such as weevils or flies, in stored cereals or crops. Products containing chlorpyrifos-methyl as an active ingredient were withdrawn from the EU in 2020 due to their potential toxic effects on human health such as genotoxic potential and developmental neurotoxicity in children (European Commission 2020; European Food Safety Authority 2019). Malathion has been used as a substitute for DDT, and there is a positive association between oral exposure levels and non-Hodgkin’s lymphoma and aggressive prostate cancer; however, the evidence is limited (Mohamed et al. 2010; World Health Organization 2016). As far as we are aware, the presence of these pesticides in SS has not been reported, with the exception of chlorpyrifos, which was detected at concentrations ranging from 0.45 to 703 ng g^-1^ and from non-detected to 0.181 ng g^-1^ (Masiá et al. 2015; Maragou et al. 2021). This may be explained by the fact that OPPs, such as malathion, can serve as a carbon and phosphorus source in activated sludge biological processes (Mohamed et al. 2010). Published data show that malathion has an affinity for adsorbing onto the surface of microplastics (Wang et al. 2020).

##### Pyrethroid pesticides

PYRs in SS samples were evaluated because they are commonly used in agriculture, veterinary, and domestic fields to control insects (Bragança et al. 2018). PYRs were not detected in samples B, E, and G. Only one previous study has reported the presence of PYRs in SS, with bifenthrin, λ-cyhalothrin, and deltamethrin detected at concentrations ranging from < 0.005 to 0.0800 mg/kg dw, < 0.01 to 0.471, and < 0.0005 to 0.171 mg/kg dw, respectively (Maragou et al. 2021). However, PYR residues have been reported in effluents and influents of WWTPs (Weston et al. 2013; Firouzsalari et al. 2019), as well as in soil (Ariyani et al. 2020; Deng et al. 2020). These pesticides are known for their affinity for OM and low water solubility (Cycon and Piotrowska-Seget 2016; Horton et al. 2018). For instance, Ziajahromi et al. (2018) described the sorption of bifenthrin to organic carbon in river water and polyethylene microparticles present in the soil as a process that reduces the bioavailability and toxicity of this compound. However, it has been suggested that microplastics can act as vectors for the uptake of pollutants by organisms (Horton et al. 2018). In addition, soil bacteria, such as *Bacillus* sp., can degrade several PYRs into phenoxybenzoic acid (3-PBA), the general metabolic product of these pesticides. 3-PBA is known for its endocrine-disrupting activity and can be toxic to non-target organisms (Chen et al. 2012; Cycon and Piotrowska-Seget 2016). Moreover, seeds of *Cucumis sativus* (*C. sativus*) exposed to cypermethrin, deltamethrin, and λ-cyhalothrin and 3-PBA for seven days showed that certain PYRs can impact the germination and early growth of plants. Exposure to 3-PBA negatively impacted the seed germination of *C. sativus*. Cypermethrin affected the roots, shoots, and leaves, while deltamethrin only affected root length. Chlorophyll and total carotenoid contents increased with exposure to cypermethrin and deltamethrin exposure, while λ-cyhalothrin had no statistically significant effects on germination, seedling development, or pigment content (Bragança et al. 2018).

##### Organophosphate esters

OPEs are a class of organophosphorus flame retardants that have been extensively used as alternatives to halogenated flame retardants (PBDEs) in plastics, electronic equipment, and textiles (Gao et al. 2016; Liang and Liu 2016; Wang et al. 2019). Their presence has been described in various environmental matrices, such as water, soil, sediments, air, biota, and human samples (Wang et al. 2018; Chokwe and Okonkwo 2019). In 2005, Marklund et al. estimated that 49% of the total annual amount of OPEs received by Swedish WWTPs were degraded, 50% were emitted in the effluents, and 1% ended up in the sludge (Marklund et al. 2005). Furthermore, in a typical WWTP, hydrophobic interactions play an important role in the sorption of OPEs to activated sludge and their subsequent biodegradation (Liang and Liu 2016). The removal of OPEs is favored under aerobic conditions, as the enzyme activity is higher and oxidation reactions are more likely to occur. Under anaerobic conditions, triphenyl OPEs (e.g., TPhP) are more likely to biodegrade (> 70%) than chlorinated OPEs (e.g., TCEP) and alkyl OPEs (e.g., TiBP) (Yang et al. 2021). Within the group of OPEs targeted in the present study, four compounds (TiBP, TPhP, TEHP, and TBEP) were detected in four SS samples and were quantified in three samples (B, E, and F). Sample F exhibited high levels of total OPEs (28.3 ± 0.013 μg g^-1^ dw; Fig. 2S in the Supporting Information). This was followed by sample E (2.05 ± 0.012 μg g^-1^ dw; Fig. 3S in the Supporting Information) and sample B (1.06 ± 0.19 μg g^-1^ dw). TiBP (26.8 ± 0.013 μg g^-1^ dw) was the most abundant compound, possibly due to its structure, which includes branched hydrocarbon chains. This structural feature increases its resistance to biodegradation (Liang and Liu 2016). A survey conducted from 2008 to 2014 investigated 14 OPEs in the sludge of eight Chinese WWTPs. TEHP and TCP were the predominant congeners found, with mean concentrations of 233 and 137 μg/kg, respectively (Gao et al. 2016). Concentrations of OPEs gradually increased over time, which is believed to be directly linked to the increased consumption of these compounds. Recently, Wang et al. (2019) analyzed 75 SS samples from 67 WWTPs in the USA. They found a median concentration of 1290 ng g^-1^ dw (∑20 OPEs). The most abundant congeners detected were TBEP and TEHP, with mean concentrations ranging from undetectable to 28,300 ng g^-1^ dw and 26.5 to 857 ng g^-1^ dw, respectively. Thus, the potential for the accumulation of OPEs in SS and their migration from soil to crops should be considered. Organic pollutants can be taken up by plants through active and passive mechanisms, which depend on the physicochemical properties and concentrations of the compounds in the surrounding environment (Gong et al. 2020). The study conducted by Gong et al. investigated the accumulation, translocation, and transformation of TCEP, TnBP, and TPhP, as well as their diester hydrolysis metabolites (namely bis(2-chloroethyl) phosphate (BCEP), di-n-butyl phosphate (DnBP), and diphenyl phosphate (DPhP)), in wheat (*Triticum aestivum* L.) plants through hydroponic experiments. TnBP, DnBP, TPhP, and DPhP primarily accumulated in the roots, while TCEP and BCEP were mainly transported to the shoots due to their hydrophilic nature. Moreover, the hydrolysis of OPEs occurs in the cell wall, so organophosphate diesters are more stable in plants than organophosphate triesters. Therefore, the possible biological effects of these compounds on the environment deserve more attention (Gong et al. 2020).

##### Persistent organic pollutants

The POPs of interest in this study belong to the chemical families of PCBs, PBDEs, OCPs, and PAHs. Even at low concentrations, these chemicals can accumulate in the food chain and cause harmful effects on both human health (e.g., carcinogenic, immunological, reproductive, and developmental effects) and the environment (Pozo et al. 2020). Among the four PCB congeners analyzed, only PCB 118 was detected and quantified in sample A, at a concentration of 0.302 ± 0.056 μg g^-1^ dw, which is below the EU limit proposed for these compounds (0.8 mg kg^-1^ dw, as shown in Fig. 4S of the Supporting Information). Additionally, the concentration in sample A was below the range of concentrations documented in Egyptian, which ranges from 897 to 2350 μg/kg, dw, as reported by Barak et al. in 2017.

Concerning PBDEs, three congeners were detected in four samples: BDE 28 (0.128 ± 0.027 μg g^-1^ dw; B: < LOQ), BDE 99 (C:0.0540 ± 0.012 μg g^-1^ dw; F: < LOQ), and BDE 183 (A < LOQ). BDE 99 is the primary component of the penta-BDE formulation, whereas BDE 183 is the primary component of octa-BDE. Both commercial PBDE formulations are listed in *Annex A (Elimination)* of the Stockholm Convention (UNEP 2009). The presence of certain PBDEs congeners in the SS samples has been documented in the scientific literature (Sánchez-Brunete et al. 2009; Wang et al. 2013; Zhao et al. 2022, 2023). For instance, six congeners were found in 19 SS samples collected from WWTPs in the province of Madrid, with concentrations ranging from 20.9 to 736.9 ng g^-1^ dw (Sánchez-Brunete et al. 2009). In another study of Spanish SS samples, the concentration of ∑$\mathrm{P}$PBDEs ranged from 16.2 to 45.6 ng g^-1^ dw (Martínez-Moral and Tena 2014). Owing to their flame retardant properties, PBDEs have been widely used in plastic production (Gaylor et al. 2013). PBDEs and plastic polymers do not bind chemically, allowing these compounds to migrate into the surrounding environment and (bio)accumulate. In addition, these compounds are lipophilic and are prone to causing adverse effects on the endocrine system, especially in the regulation of thyroid hormones (Martínez-Moral and Tena 2014). Moreover, Gaylor et al. (2013) conducted a study on the absorption of PBDEs by earthworms (*Eisenia fetida*) in artificial soil. The soil was supplemented with anaerobically digested biosolids, composted biosolids, penta-BDE-spiked polyurethane foam microparticles, and penta-BDE-spiked artificial soil. The study found that *E. fetida* accumulated penta-PBDE from both biosolids and plastic waste, indicating the potential for PBDEs to bioaccumulate and transfer through food chains.

The accumulation of OCPs compounds in SS stems from their persistent nature, adsorption capacity for solid particles, and hydrophobic character (Barakat et al. 2017; Ademoyegun et al. 2020). In this study, α-endosulfan was the only analyte detected in all samples, with concentrations ranging from 0.110 ± 0.007 to 0.571 ± 0.037 μg g^-1^ dw. This OCP is one of two technical endosulfan isomers that were banned worldwide during the 1970s and 1980s (Somtrakoon and Pratumma 2012). However, in 2017, α-endosulfan was detected in SS samples from Egypt (ranging from 3.24 to 76.8 μg kg^-1^) (Barakat et al. 2017), and in 2020, it was found in South Africa with concentrations ranging from 8 to 99 ng g^-1^ dw (Ademoyegun et al. 2020).

Regarding PAHs, the EU sludge directive recommends that ∑PAHs should not exceed 6 mg kg^-1^ dw in sludge used for agricultural reclamation. The presence of PAHs in SS samples has been extensively reported worldwide. For example, in Taiwan, the concentration of ∑16 PAHs was found to be 0.5332–1.0666 µg kg^-1^ dw (Chen et al. 2019). In Poland, the concentration ranged from 8625 to 13,833 μg kg^-1^ dw (Tomczyk et al. 2020). In Italy, the concentration ranged from 0.01 to 1.48 mg kg^-1^ dw and 0.04 to 3.91 mg kg^-1^ dw (Suciu et al. 2015). In this study, sample C exhibited the highest levels of total PAHs (0.479 ± 0.0044 μg g^-1^ dw; Fig. 5S from the Supporting Information). It was followed by sample G (0.401 ± 0.014 μg g^-1^ dw) and sample E (0.0418 ± 0.00003 μg g^-1^ dw). FLU (a 3-ring PAH) was the most abundant compound (C: 0.435 ± 0.0042 μg g^-1^ dw; G: 0.401 ± 0.014 μg g^-1^ dw), followed by BaP (a 5-ring PAH) and DBA (a 5-ring PAH), both prioritized for their teratogenic, mutagenic, and carcinogenic characteristics (Wołejko et al. 2018). Samples A, B, D, and F had no detectable congeners.

Overall, the results showed that the developed QuEChERS methodology can be coupled with chromatographic techniques such as GC-FPD, GC-ECD, GC-MS, and LC-PDA-FLD to monitor a wide range of chemical classes in SS. The QuEChERS extraction method enables the use of small amounts of sample and solvent, reducing sample preparation time, and facilitating the use of eco-friendly solvents, especially when compared to classical methods such as Soxhlet, all while demonstrating high efficiency (Perestrelpo et al. 2019). The complexity of the matrix presents challenges in sample preparation for the development of reliable analytical methods, with the cleanup step being crucial. The levels of pollutants in the SS varied among different samples, which could be attributed to both the composition of each wastewater and the distinct characteristics of each treatment system. Sorption–desorption phenomena determine the fate of organic micropollutants in WWTPs. Moreover, the interaction of organic pollutants with activated sludge influences their bioavailability to microorganisms and the subsequent biodegradation during biological treatment (Madrid et al. 2020). The lack of data on SS contamination by organic pollutants, possibly due to the complexity of the matrix, and the potential negative effects on the environment, such as the possibility of migration from soils to crops, are reflected in the challenges of implementing legislation (Calderón-Preciado et al. 2013; Suciu et al. 2015). On the one hand, the reuse of SS allows for the substitution of synthetic fertilizers and the utilization of waste that is abundant in organic nutrients. On the other hand, this practice can pose risks to both the environment and human health, as micropollutants have the potential to accumulate in SS (Martín-Pozo et al. 2019; Madrid et al. 2020). Hence, more studies are needed in the future to evaluate the exposure of SS to soil, particularly those that encompass different chemical families, as they provide a more realistic representation.

## Conclusion

The developed method, based on the QuEChERS approach, allows for the simultaneous extraction and analysis of 42 target analytes from complex SS matrices. Purification of the extracts was a critical step because commercial d-SPEs did not prove to be the most efficient. Therefore, CB and Z-Sep had to be added. Thus, it was possible to obtain clean extracts for analysis by gas chromatography. The procedure provided good recoveries and reproducibility, enabling the quantification of all target analytes while minimizing the time needed for sample preparation. Accurate quantification was achieved by using calibration standards that were matched to the matrix. The method yielded satisfactory performance, with a mean recovery ranging from 50 to 126%, and a reproducibility ≤ 21% (RSD). The protocol was successfully applied to seven SS samples, and at least two target compounds were detected in each sample. Of the 42 targeted analytes, 16 were detected at concentrations ranging from 0.0420 to 26.8 μg g^-1^ dw. Of these, eight are listed as POPs and were detected with concentrations ranging from < LOQ to 0.571 µg g^-1^ dw. The pesticide α-endosulfan was quantified in all samples, with concentrations ranging from 0.110 to 0.571 µg g^-1^ dw. Even if the detected compounds meet the legal limits (when applicable) or occur in relatively low concentrations, it should not be forgotten that little is known about the toxic effects that such a mixture may have on human health. For the past 36 years, the Sewage Sludge Directive has aimed at the correct use of SS in agriculture, while safeguarding soil, vegetation, animals, and humans. Furthermore, under the European Green Deal, there is a need to move from a linear economic model to a circular one that is capable of increasing resource efficiency. Consequently, sludge application to agricultural soils may increase in the coming years, particularly in semi-arid areas. In addition, all products used daily, such as pharmaceuticals, can end up in WWTPs and potentially accumulate in SS. Therefore, the inherent nature of the SS matrix and the dynamic behavior of pollutants in the environment make it necessary to broaden the scope of chemical compounds covered by the legislation.

### Supplementary Information

Below is the link to the electronic supplementary material.

Supplementary file1 (DOCM 459 KB)

## Data Availability

Not applicable.
