# Supplementary material for: Assessing emerging and priority micropollutants in sewage sludge: environmental insights and analytical approaches
Source: Environ Sci Pollut Res Int. 2023 Dec 12;31(2):3152–68. doi: 10.1007/s11356-023-30963-1 (PMC10791843; doi:10.1007/s11356-023-30963-1)
Supplement: Supplementary file 1 — Supplementary file1 (DOCM 459 KB) [file 11356_2023_30963_MOESM1_ESM.docm]

**Assessing Emerging and Priority Micropollutants in Sewage Sludge: Environmental Insights and Analytical Approaches**

Diana Rede^1,2^, Ivan Teixeira^1^, Cristina Delerue-Matos^1^, and Virgínia Cruz Fernandes^1,*^

^1^REQUIMTE/LAQV, Instituto Superior de Engenharia do Porto, Instituto Politécnico do Porto, Rua Dr. António Bernardino de Almeida, 431, 4249-015 Porto, Portugal

^2^Departamento de Química e Bioquímica, Faculdade de Ciências, Universidade do Porto, Rua do Campo Alegre s/n, 4169– 007 Porto, Portugal.

*Corresponding author: [vir@isep.ipp.pt](mailto:vir@isep.ipp.pt)

**S.1 Materials and Methods**

## **
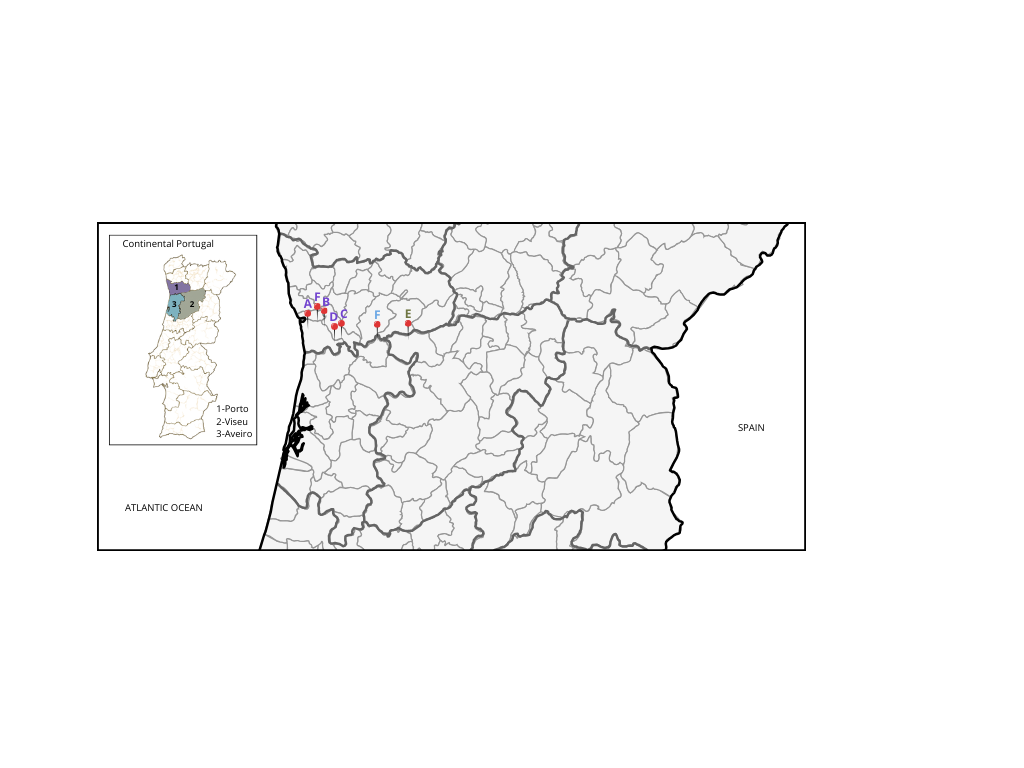
S.1.1 Sampling Sites**

**Fig. 1S** Map of the sampling sites located in Continental Portugal - Sampling sites (A to F) across the Porto, Viseu and Aveiro regions (1 to 3)

**S.1.2 Physical, Chemical, and Elemental Characterization of Sewage Sludge**

The physicochemical characterization of the samples was carried out according to standardized methods. All parameters were analyzed in duplicate, and blanks were also prepared. For instance, the pH in water (pH_w_) was determined using SW-846 Test Method 9045D: Soil and Waste pH from EPA (USEPA, 1986). The measurements were performed at room temperature in a 1:2 (m/V) SS/water suspension using an electronic pH meter (micropH 2002 Crison, Spain) after 20-minute of contact time. Dry matter (DM) and organic matter (OM) content were assessed according to EN 12457-4 (CEN, 2002) and the Laboratory Guide for Conducting Soil Tests and Plant Analysis (J.B.Jones, 2001), respectively. DM was determined by weighing the difference, using a forced-draft oven (J.P. Selecta S.A 2000200), where the samples were placed overnight at 105 °C. OM was estimated using the loss-on-ignition methodology. The previous dried samples were placed into a muffle furnace (Nabertherm-B 180, Germany) at 450 °C for 4 hours. Total carbon (TOC) was determined using a Shimadzu TOC-Vscn E200V analyzer with SSM-5000A Solid Sample Module (Shimadzu Corporation, Kyoto, Japan).

For the determination of mineral and metal content, samples were digested using HNO_3_, in open vessels covered with a watch glass and heated conventional (APHA, 1992). After cooling, the volume of the digested samples was adjusted to 10 mL using deionized water. The concentrations of Co, Mo, Hg, Cd, Ni, Pb, Cr, Cu, Zn, Li, Be, V, As, Se, Rb, Sr, Rh, Sb, Cs, Ba, Ir, Tl, Mg, Ca, Mn, and Fe were determined using Ion Coupled Plasma Mass Spectrometry (ICP-MS) performed with a Thermo Fisher Scientific (Bremen, Germany) iCAP™ Q instrument, which was equipped with a MicroMist™ nebulizer (Glass Expansion, Port Melbourne Vic, Australia). The instrument also included a Peltier-cooled baffled cyclonic spray chamber, a standard quartz torch, and a two-cone interface design with Ni sample and skimmer cones., High-purity argon (99.9997%; Praxair, Portugal) was used as the carrier gas. Prior to use, all the labware was properly soaked in 10% HNO_3_ and rinsed with deionized water.

**S.1.3 Chromatographic Analysis**

**PAHs** were quantified using an LC system (Shimadzu Corporation, Kyoto, Japan), equipped with an LC-20AD pump, DGU-20AS degasser, and photodiode array SPD-M20A, as well as fluorescence RF-10AXL detectors. The LC system was operated using LC Solution Shimadzu software. Chromatographic separation was performed at 20 ± 1 °C by injecting 15.0 μL of sample extracts onto a NUCLEOSIL® 100-5 C18 PAH column (150 x 4.0 mm and 5 μm particle size) from Macherey–Nagel (Duren, Germany), with a flow rate of 0.8 mL min^-1^. The chromatographic conditions consisted of a mixture of 50% ACN and 50% ultrapure water. Then, the elution gradient was increased to 100% over 15 minutes, and these conditions were maintained for an additional 13 minutes (Oliveira et al., 2015).

**OPPs** and **OPEs** were determined using a Shimadzu GC-FPD operated by the Shimadzu GC Solution software. The injector and detector temperatures were maintained at 250 and 290 °C, respectively. 1.0 µL of the sample solution was injected in splitless mode on a capillary column TRB-5 (30 m in length, 0.32 mm in inner diameter (i.d.), with a 0.25 µm film thickness, manufactured by Teknokroma, Barcelona, Spain). The GC oven temperature was programmed as follows: 100 °C for 1 min, ramped at 20 °C min^-1^ to 150 °C, held for 1 min, ramped at 2 °C min^-1^ to 180 °C, held for 2 min, and finally ramped at 20 °C min^-1^ to 290 °C, and held at the final temperature for 5 min. Helium (purity 99.99% from Linde Sogás) was used at a pressure of 83.6 KPa, linear velocity of 25.4 cm s^-1^, and flow rate of 1 mL min^-1^.

The analysis of **PBDEs**, **PCBs**, **OCPs**, and **PYRs** was performed in splitless mode with a 2 μL injection volume using a GC-ECD operated by GC Solution Shimadzu software. A capillary column, Zebron-5MS (30 m × 0.25 mm × 0.25 μm), from Phenomenex Spain, was used. The oven temperature was initially set at 40 °C (held for 1 min), then increased to 120 °C at a rate of 15 °C min^-1^ per minute and held for 1 min. After the temperature was increased to 200 °C at a rate of 10 °C min^-1^ (held for 1 min), it was further increased to 290 °C at a rate of 7 °C min^-1^ and maintained for 10 min. The injector and detector temperatures were set at 250 °C and 300 °C, respectively. The flow rate of the helium carrier gas (Nippon Gases, Portugal) was set at 1.3 mL min^-1^ with a linear velocity of 32.7 cm s^-1^, and the nitrogen make-up gas (Nippon Gases, Portugal) at a rate of 30 mL min^-1^.

GC-MS analyses were performed using a Thermo Fisher Scientific instrument (Waltham, USA) coupled with an ion trap mass detector (Thermo Polaris). The analytes were separated on a Zebron-5ms capillary column (30 m × 250 μm i.d., 0.25 μm thickness, Phenomenex, Spain). The oven temperature was initially maintained at 40 °C for 1 minute, and then the temperature was increased to 290 °C as follows: 120 °C at a rate of 15 °C per minute, held for 1 minute; 150 °C (10 °C per minute), held for 1 minute; 180 °C (10 °C per minute), held for 1 minute; 200 °C (10 °C per minute), held for 1 minute; and 290 °C (10 °C per minute), held for 10 minutes. The helium carrier gas (purity ≥ 99.999%) was maintained at a constant flow rate of 1.0 mL/min. The temperatures of the inlet, ion source, and transfer line were maintained at 250 °C. The injection volume of 1 μL entered the GC-MS system in splitless mode. The mass spectrometer was operated with electron impact (eI) ionization (-70 eV) with a filament current of 200 μA in the SIM and tandem MS modes. The retention time, precursor, and fragment ions are listed in Table S1. At least three fragment ions were monitored for each compound. To maximize the detector signal, the most abundant and characteristic ion in the spectrum was selected for confirmation.

**Table S1:** SIM program used to confirm the OPPs, OPEs, PCBs, PBDEs, OCPs, and PYRs detected in the SS samples.

| Compound | m/z | Retention time (min) | Dwell time (ms) |
| --- | --- | --- | --- |
| TiBP | 99,155, 211, 265 | 13.16 | 99 |
| Chlorpyrifos-methyl | 109, 125, 197, 286 | 19.07 | 286 |
| Malathion | 93, 99, 125, 158, 173 | 20.01 | 173 |
| α-endosulfan | 195, 241, 265, 277 | 21.82 | 195 |
| PCB118 | 145, 184, 219, 254, 256, 326 | 23.28 | 326 |
| BDE 28 | 168, 223, 247, 406 | 23.42 | 406 |
| TPhP | 77, 169, 215, 233, 326 | 24.66 | 215 |
| TEHP | 57, 99, 113, 211, 323 | 24.91 | 99 |
| BDE 99 | 404, 564, 566 | 27.78 | 404 |
| Cypermethrin | 91, 127, 152, 163 | 28.11 | 163 |
| Deltamethrin | 77, 93, 172, 209, 253 | 30.13 | 253 |
| BDE 183 | 561, 564, 723 | 34.17 | 561 |

**S.2 Results**

**S.2.1 Physical, Chemical, and Elemental Characterization of Sewage Sludge Samples**

**Table S2:** Physicochemical properties, heavy metals, and other trace elements content of SS samples (n=2) (mean ± SD) (ND = Not detected).

| **Parameter** | **Sewage Sludge Samples** | | | | | | |
| --- | --- | --- | --- | --- | --- | --- | --- |
|  | A | B | C | D | E | F | G |
| **pH** | 8.42 | 6.08 | 7.98 | 6.61 | 6.08 | 6.93 | 7.38 |
| **DM (%)** | 24.1 | 15.5 | 17.0 | 14.4 | 13.4 | 20.7 | 19.8 |
| **OM (% dw)** | 68.7 | 85.1 | 74.1 | 85.8 | 81.5 | 85.2 | 73.5 |
| ***Primary nutrients*** |  |  |  |  |  |  |  |
| TOC (%) | 36.8 | 40.5 | 32.8 | 27.3 | 16.8 | 44.3 | 35.2 |
| ***Secondary nutrients*** |  |  |  |  |  |  |  |
| Ca (mg kg^-1^dw) | 12,473 | 2,857 | 10,541 | 10,162 | 7,579 | 12,265 | 13,016 |
| Mg (mg kg^-1^dw) | 1,619 | 1,129 | 2,743- | 2,927 | 1,799 | 2,242 | 2,779 |
| ***Micro nutrients*** |  |  |  |  |  |  |  |
| Co (mg kg^-1^dw) | 2.35 | 1.26 | 60.2 | 2.31 | 1.57 | 1.56 | 2.84 |
| Mo (mg kg^-1^dw) | 3.23 | 2.14 | 4.93 | 3.75 | 2.88 | 3.06 | 4.00 |
| Mn (mg kg^-1^dw) | 105 | 43.6 | 643 | 75.2 | 108 | 71.6 | 127 |
| Fe (mg kg^-1^dw) | 3,957 | 1,412 | 8,856 | 2,867 | 3,041 | 2,247 | 6,994 |
| ***Heavy metals*** |  |  |  |  |  |  |  |
| Hg (mg kg^-1^dw) | 0.060 | 0.050 | 0.110 | 0.170 | 0.190 | 0.050 | 0.210 |
| Cd (mg kg^-1^dw) | 0.730 | 0.290 | 7.89 | 0.790 | 0.660 | 0.810 | 1.10 |
| Ni (mg kg^-1^dw) | 59.5 | 16.5 | 110 | 29.7 | 14.2 | 11.0 | 20.1 |
| Pb (mg kg^-1^dw) | 27.8 | 8.51 | 38.3 | 22.8 | 14.9 | 20.6 | 32.9 |
| Cr (mg kg^-1^dw) | 125 | 12.3 | 32.2 | 27.7 | 13.9 | 12.7 | 25.2 |
| Cu (mg kg^-1^dw) | 172 | 115 | 246 | 181 | 140 | 179 | 269 |
| Zn (mg kg^-1^dw) | 906 | 238 | 2187 | 634 | 453 | 689 | 935 |
| ***Other trace elements*** |  |  |  |  |  |  |  |
| Li (mg kg^-1^dw) | 7.80 | 1.44 | 7.01 | 3.55 | 8.37 | 3.67 | 16.9 |
| Be (mg kg^-1^dw) | 0.450 | 0.190 | 2.62 | 0.180 | 0.550 | 0.330 | 1.34 |
| V (mg kg^-1^dw) | 6.71 | 1.52 | 9.62 | 5.56 | 4.42 | 5.32 | 8.69 |
| As (mg kg^-1^dw) | 5.07 | 2.22 | 13.3 | 6.50 | 8.24 | 5.59 | 8.62 |
| Se (mg kg^-1^dw) | 3.17 | 2.89 | 6.80 | 2.62 | 2.54 | 2.53 | 3.80 |
| Rb (mg kg^-1^dw) | 8.67 | 3.72 | 8.95 | 6.63 | 1.38 | 5.42 | 19.8 |
| Sr (mg kg^-1^dw) | 66.7 | 14.4 | 67.8 | 106.4 | 26.2 | 42.7 | 37.7 |
| Rh (mg kg^-1^dw) | ND | ND | ND | ND | ND | ND | 0.860 |
| Sb (mg kg^-1^dw) | 0.120 | 0.050 | 0.250 | 0.150 | 0.060 | 0.560 | 0.060 |
| Cs (mg kg^-1^dw) | 2.06 | 0.850 | 2.61 | 1.99 | 5.22 | 3.33 | 4.45 |
| Ba (mg kg^-1^dw) | 232 | 40.8 | 303 | 99.3 | 71.1 | 110 | 154 |
| Ir (mg kg^-1^dw) | ND | ND | 0.190 | ND | ND | 1.33 | 1.34 |
| Tl (mg kg^-1^dw) | 0.110 | 0.0500 | 0.310 | 0.0800 | 0.140 | 0.150 | 0.210 |

**S.2.2 Levels of Organic Pollutants in Sewage Sludge Samples**

**Table S3:** Concentrations (μg g^-1^ dw) of OPPs, OPEs, PBDEs, PCBs, OCPs, PYRs, and PAHS in sewage sludge collected from municipal wastewater treatment plants located in the north of Portugal (mean values, n=6; ND: not detected; <LOQ: below method quantification limit).

| Target analyte | | SS samples | | | | | | |
| --- | --- | --- | --- | --- | --- | --- | --- | --- |
|  |  | A | B | C | D | E | F | G |
| OPPs | Dimethoate | ND | ND | ND | ND | ND | ND | ND |
|  | Chlorpyrifos methyl | ND | ND | <LOQ | ND | ND | ND | ND |
|  | Parathion methyl | ND | ND | ND | ND | ND | ND | ND |
|  | Malathion | ND | ND | ND | <LOQ | ND | ND | ND |
|  | Chlorpyrifos | ND | ND | ND | ND | ND | ND | ND |
|  | Chlorfenvinphos | ND | ND | ND | ND | ND | ND | ND |
|  | ∑6 OPP |  |  |  |  |  |  |  |
| OPEs | TiBP | ND | ND | ND | ND | ND | 26.8±0.013 | ND |
|  | TnBP | ND | ND | ND | ND | ND | ND | ND |
|  | TCEP | ND | ND | ND | ND | ND | ND | ND |
|  | TPhP | ND | ND | ND | <LOQ | 2.05±0.012 | ND | ND |
|  | TBEP | ND | 1.06±0.19 | ND | ND | ND | ND | ND |
|  | TEHP | ND | ND | ND | ND | ND | 1.53±0.023 | ND |
| . | TCP | ND | ND | ND | ND | ND | ND | ND |
|  | ∑7 OPE |  | 1.06±0.19 |  |  | 2.05±0.012 | 28.3±0.068 |  |
| PCBs | PCB 28 | ND | ND | ND | ND | ND | ND | ND |
|  | PCB 118 | 0.302±0.056 | ND | ND | ND | ND | ND | ND |
|  | PCB 153 | ND | ND | ND | ND | ND | ND | ND |
|  | PCB 180 | ND | ND | ND | ND | ND | ND | ND |
|  | ∑4 PCB | 0.302±0.056 |  |  |  |  |  |  |
| PBDEs | BDE 28 | 0.128±0.027 | <LOQ | ND | ND | ND | ND | ND |
|  | BDE 47 | ND | ND | ND | ND | ND | ND | ND |
|  | BDE 100 | ND | ND | ND | ND | ND | ND | ND |
|  | BDE 99 | ND | ND | 0.054±0.012 | ND | ND | <LOQ | ND |
|  | BDE 154 | ND | ND | ND | ND | ND | ND | ND |
|  | BDE 153 | ND | ND | ND | ND | ND | ND | ND |
|  | BDE 183 | <LOQ | ND | ND | ND | ND | ND | ND |
|  | ∑7 BDE | 0.128±0.027 |  | 0.054±0.012 |  |  |  |  |
| OCPs | o-HCH | ND | ND | ND | ND | ND | ND | ND |
|  | α-Endosulfan | 0.110±0.007 | 0.170±0.005 | 0.490±0.084 | 0.571±0.037 | 0.366±0.010 | 0.282±0.019 | 0.286±0.022 |
|  | p,p´-DDE | ND | ND | ND | ND | ND | ND | ND |
|  | p,p´-DDD | ND | ND | ND | ND | ND | ND | ND |
|  | Dieldrin | ND | ND | ND | ND | ND | ND | ND |
|  | ∑5 OCP | 0.110±0.007 | 0.170±0.005 | 0.490±0.084 | 0.571±0.037 | 0.366±0.010 | 0.282±0.019 | 0.286±0.022 |
| PYRs | Bifenthrin | ND | ND | ND | ND | ND | ND | ND |
|  | λ-Cyhalothrin | ND | ND | ND | ND | ND | ND | ND |
|  | Cypermethrin | 0.0870±0.012 | ND | 0.0573±0.011 | ND | ND | <LOQ | ND |
|  | Fenvalerate | ND | ND | ND | ND | ND | ND | ND |
|  | Deltamethrin | 0.254±0.0560 | ND | ND | <LOQ | ND | ND | ND |
|  | ∑5 PYR | 0.341±0.068 |  | 0.0573±0.011 |  |  |  |  |
| PAHs | Fluoranthene | ND | ND | 0.435±0.0042 | ND | ND | ND | 0.401±0.0143 |
|  | Benzo[b+j]fluoranthen | ND | ND | ND | ND | ND | ND | ND |
|  | Benzo[a]pyrene | ND | ND | 0.0442±0.0002 | ND | 0.0418±0.00003 | ND | ND |
|  | Dibenzo[a,l]pyrene | ND | ND | ND | ND | ND | ND | ND |
|  | Dibenz[a,h]anthracene | ND | ND | <LOQ | ND | ND | ND | <LOQ |
|  | Benzo[ghi]perylene | ND | ND | ND | ND | ND | ND | ND |
|  | Indeno[1,2,3‐cd]pyrene | ND | ND | ND | ND | ND | ND | ND |
|  | ∑9 PAH |  |  | 0.479±0.0044 |  | 0.0418±0.00003 |  | 0.401±0.0143 |
| Total pollutant load per SS sample (μg g^-1^ dw) | | 0.881 | 1.26 | 2.25 | 0.571 | 2.46 | 28.6 | 0.687 |
| Total POP load per SS sample (μg g^-1^ dw) | | 0.540 | 0.170 | 1.02 | 0.571 | 0.408 | 0.282 | 0.687 |
| Number of pollutants detected per SS sample | | 6 | 3 | 7 | 4 | 3 | 5 | 2 |

**Table S4:** Literature-reported concentrations for OPPs, OPEs, PBDEs, PCBs, OCPs, PYRs, and PAHS in sewage sludge.

| **Target analyte** | | **Concentration** | **Analytical procedure** | **Recovery (%)** | **Method sensitivity** | **Reference** |
| --- | --- | --- | --- | --- | --- | --- |
| OPPs | Chlorfenvinfos | ND | QuEChERS-LC-MS/MS | 91 | LOQ=5 ng g^-1^ | (Masiá et al., 2015) |
|  | Chlorpyrifos | 0.45-703 ng g^-1^ |  | 51 | LOQ=0.1 ng g^-1^ |  |
|  |  | ND-0.181 |  | - | LOD=25 ng g^-1^  LOQ=50 ng g^-1^ | (Maragou et al., 2021) |
|  | Chlorpyrifos Methyl | ND |  | - | LOD=25 ng g^-1^  LOQ=50 ng g^-1^ | (Maragou et al., 2021) |
|  | Dimethoate | ND |  | 90 | LOQ=5 ng g^-1^ | (Masiá et al., 2015) |
|  | Malathion | ND |  | 101 | LOQ=5 ng g^-1^ | (Masiá et al., 2015) |
|  | Parathion-methyl | ND |  | 40 | LOQ=10 ng g^-1^ | (Masiá et al., 2015) |
| PYRs | Bifenthrin | ND-0.0800 mg/kg dw |  | **-** | LOD=5 ng g^-1^  LOQ=10 ng g^-1^ | (Maragou et al., 2021) |
|  | λ-cyhalothrin | ND-0.471 mg/kg dw |  | **-** | LOD=10 ng g^-1^  LOQ=50 ng g^-1^ | (Maragou et al., 2021) |
|  | deltamethrin | ND-0.171 mg/kg dw |  | **-** | LOD=5 ng g^-1^  LOQ=10 ng g^-1^ | (Maragou et al., 2021) |
|  | ß-cyfluthrin | ND |  | **-** | LOD=50 ng g^-1^  LOQ=100 ng g^-1^ | (Maragou et al., 2021) |
| OPEs | TPrP | <LOD | SLE-SPE-LC-MS/MS | 102.8 | LOD=0.41 μg/kg dw | (Gao et al., 2016) |
|  | TCEP | <LOD-200 μg/kg dw |  | 64.6 | LOD=8.72 μg/kg dw |  |
|  |  | ND-317 ng/g dw | SLE-SPE-HPLC-MS/MS | 67.0 | MDL=0.64 ng/g dw | (Wang et al., 2019) |
|  | TPhP | 4.40-66.8 μg/kg dw | SLE-SPE-LC-MS/MS | 100.1 | LOD=2.02 μg/kg dw | (Gao et al., 2016) |
|  |  | ND-1170 ng/g dw | SLE-SPE-HPLC-MS/MS | 65.4 | MDL=0.73 ng/g dw | (Wang et al., 2019) |
|  | TBEP | 6.30-281 μg/kg dw | SLE-SPE-LC-MS/MS | 144.0 | LOD=0.50 μg/kg dw | (Gao et al., 2016) |
|  | TEHP | 46.0-1200 μg/kg dw | SLE-SPE-LC-MS/MS | 69.6 | LOD=1.21 μg/kg dw |  |
|  |  | 26.5-857 ng/g dw | SLE-SPE-HPLC-MS/MS | 75.0 | MDL=1.62 ng/g dw | (Wang et al., 2019) |
|  | TnBP | 1.20-286 μg/kg dw | SLE-SPE-LC-MS/MS | 99.9 | LOD=0.72 μg/kg dw | (Gao et al., 2016) |
|  |  | ND-6080 ng/g dw | SLE-SPE-HPLC-MS/MS | 65.0 | MDL=1.70 ng/g dw | (Wang et al., 2019) |
|  | TiBP | LOD-474 μg/kg dw | SLE-SPE-LC-MS/MS | 113.4 | LOD=0.63 μg/kg dw | (Gao et al., 2016) |
|  |  | ND-30.7 ng/g dw | SLE-SPE-HPLC-MS/MS | - | MDL=0.15 ng/g dw | (Wang et al., 2019) |
| PCBs | PCB 28 | 452-2380 μg/kg dw | Soxhlet-GC-MS | 94-105 | MDL=0.012 μg/kg | (Barakat et al., 2017) |
|  | PCB 118 | 897-2350 μg/kg dw |  |  | MDL=0.012 μg/kg |  |
|  | PCB 153 | 842-2150 μg/kg dw |  |  | MDL=0.012 μg/kg |  |
|  | PCB 180 | 362-722 μg/kg dw |  |  | MDL=0.012 μg/kg |  |
| PBDEs  . | BDE 28 | ND | MSPD-GC-MS | 90.7-94.4 | LOD=0.06 ng/g  LOQ=0.18 ng/g | (Sánchez-Brunete et al., 2009) |
|  |  | 0.30-46.9 ng g^-1^ dw | sPLE-MEPS-GC-MSMS | 97 | MLOD=0.02 ng g^-1^ | (Martínez-Moral and Tena, 2014) |
|  | BDE47 | ND | MSPD-GC-MS | 100.5-101.6 | LOD=0.08 ng/g  LOQ=0.24 ng/g | (Sánchez-Brunete et al., 2009) |
|  |  | 3.6-497 ng g^-1^ dw | sPLE-MEPS-GC-MSMS | 94 | MLOD=0.02 ng g^-1^ | (Martínez-Moral and Tena, 2014) |
|  | BDE99 | ND | MSPD-GC-MS | 94.4-103.6 | LOD=0.09 ng/g  LOQ=0.28 ng/g | (Sánchez-Brunete et al., 2009) |
|  |  | 1.1-892 ng g^-1^ dw | sPLE-MEPS-GC-MSMS | 102 | MLOD=0.04 ng g^-1^ | (Martínez-Moral and Tena, 2014) |
|  | BDE100 | ND-7.6 ng/g | MSPD-GC-MS | 98.7-107.5 | LOD=0.1 ng/g  LOQ=0.35 ng/g | (Sánchez-Brunete et al., 2009) |
|  |  | 5.2-148 ng g^-1^ dw | sPLE-MEPS-GC-MSMS | 92 | MLOD=0.02 ng g^-1^ | (Martínez-Moral and Tena, 2014) |
|  | BDE153 | ND-6.3 ng/g | MSPD-GC-MS | 77.9-107.0 | LOD=0.25 ng/g  LOQ=0.8 ng/g | (Sánchez-Brunete et al., 2009) |
|  | BDE154 | ND-17.6 ng/g |  | 79.4-109.3 | LOD=0.2 ng/g  LOQ=0.68 ng/g |  |
|  |  | 2.7-83.5 ng g^-1^ dw | sPLE-MEPS-GC-MSMS | 92 | MLOD=0.02 ng g^-1^ | (Martínez-Moral and Tena, 2014) |
|  | BDE183 | ND-4.3 ng/g | MSPD-GC-MS | 77.9-98.0 | LOD=0.4 ng/g  LOQ=1.4 ng/g | (Sánchez-Brunete et al., 2009) |
| OCPs | o-HCH | 12.8-87.7 μg/kg dw | Soxhlet-GC-MS | 90-105 | MDL=0.004 μg/kg | (Barakat et al., 2017) |
|  |  | ND | SLE-GC/µECD | 93.6-104.5 | LOD=0.31 ng/g  LOQ=0.32 ng/g | (Ademoyegun et al., 2020) |
|  | α-Endosulfan | 9.62-16.4 μg/kg dw | Soxhlet-GC-MS | 90-105 | MDL=0.004 μg/kg | (Barakat et al., 2017) |
|  |  | ND-99 ng/g | SLE-GC/µECD | 91.5-99.1 | LOD=0.33 ng/g  LOQ=1.31 ng/g | (Ademoyegun et al., 2020) |
|  | p,p´-DDE | 2.13-22.2 μg/kg dw | Soxhlet-GC-MS | 90-105 | MDL=0.004 μg/kg | (Barakat et al., 2017) |
|  |  | 12-45 ng/g | SLE-GC/µECD | 98.5-107.4 | LOD=0.48 ng/g  LOQ=0.38 ng/g | (Ademoyegun et al., 2020) |
|  | p,p´-DDD | ND-7.51 μg/kg dw | Soxhlet-GC-MS | 90-105 | MDL=0.004 μg/kg | (Barakat et al., 2017) |
|  |  | 56-169 ng/g | SLE-GC/µECD | 95.6-105 | LOD=0.21 ng/g  LOQ=0.28 ng/g | (Ademoyegun et al., 2020) |
|  | Dieldrin | ND |  | 83.6-93.6 | LOD=0.43 ng/g  LOQ=0.54 ng/g |  |
| PAHs | Fluoranthene | 0.0067-0.1516 mg/kg dw | SLE-GC-MS | 93 | DL=0.0022 mg/kg dw | (Chen et al., 2019) |
|  | Benzo[a]pyrene | ND-0.0568 mg/kg dw |  | 92 | DL=0.0045 mg/kg dw |  |
|  |  | 1-1341 mg kg^-1^ dw | Soxhlet-GC-MS | 99 | - | (Suciu et al., 2015) |
|  | Dibenz[a,h]anthracene | ND-0.0434 mg/kg dw | SLE-GC-MS | 97 | DL=0.0045 mg/kg dw | (Chen et al., 2019) |
|  | Benzo[ghi]perylene | 0.0008-0.0997 mg/kg dw |  | 97 | DL=0.0033 mg/kg dw |  |
|  |  | 2-1030 mg kg^-1^ dw | Soxhlet-GC-MS | 105 | - | (Suciu et al., 2015) |
|  | Indeno[1,2,3‐cd]pyrene | ND-0.0754 mg/kg dw | SLE-GC-MS | 98 | DL=0.0046 mg/kg dw | (Chen et al., 2019) |
|  |  | 1-1310 mg kg^-1^ dw | Soxhlet-GC-MS | 68 | - | (Suciu et al., 2015) |


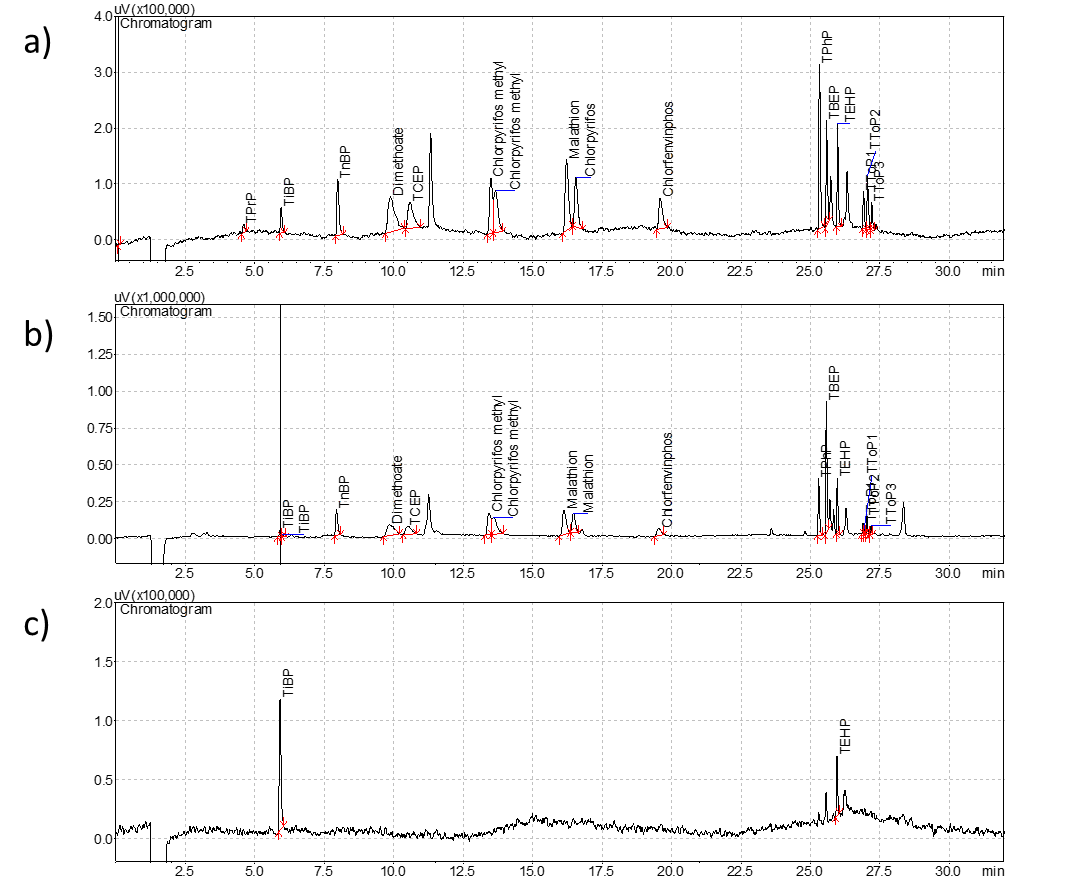


**Fig. 2S** Chromatographic data for OPPs and OPEs obtained by GC-FPD. a) Chromatogram of a matrix-matched standard solution; b) chromatogram of a reagent-only standard solution; and c) chromatogram of sample F


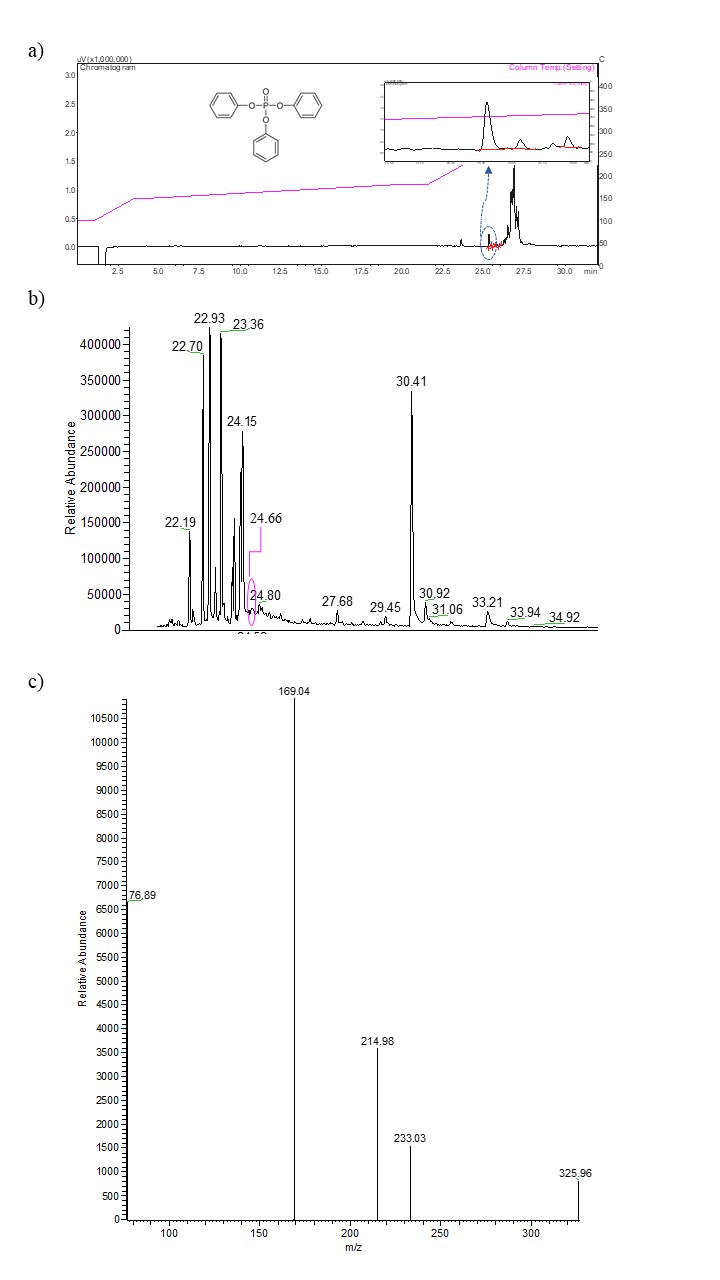


**Fig. 3S** Chromatographic information of TPhP in sample E: a) chromatogram of sample E obtained by GC-FPD, b) chromatogram of sample E obtained by GC-MS, and c) mass spectrum of TPhP in sample E


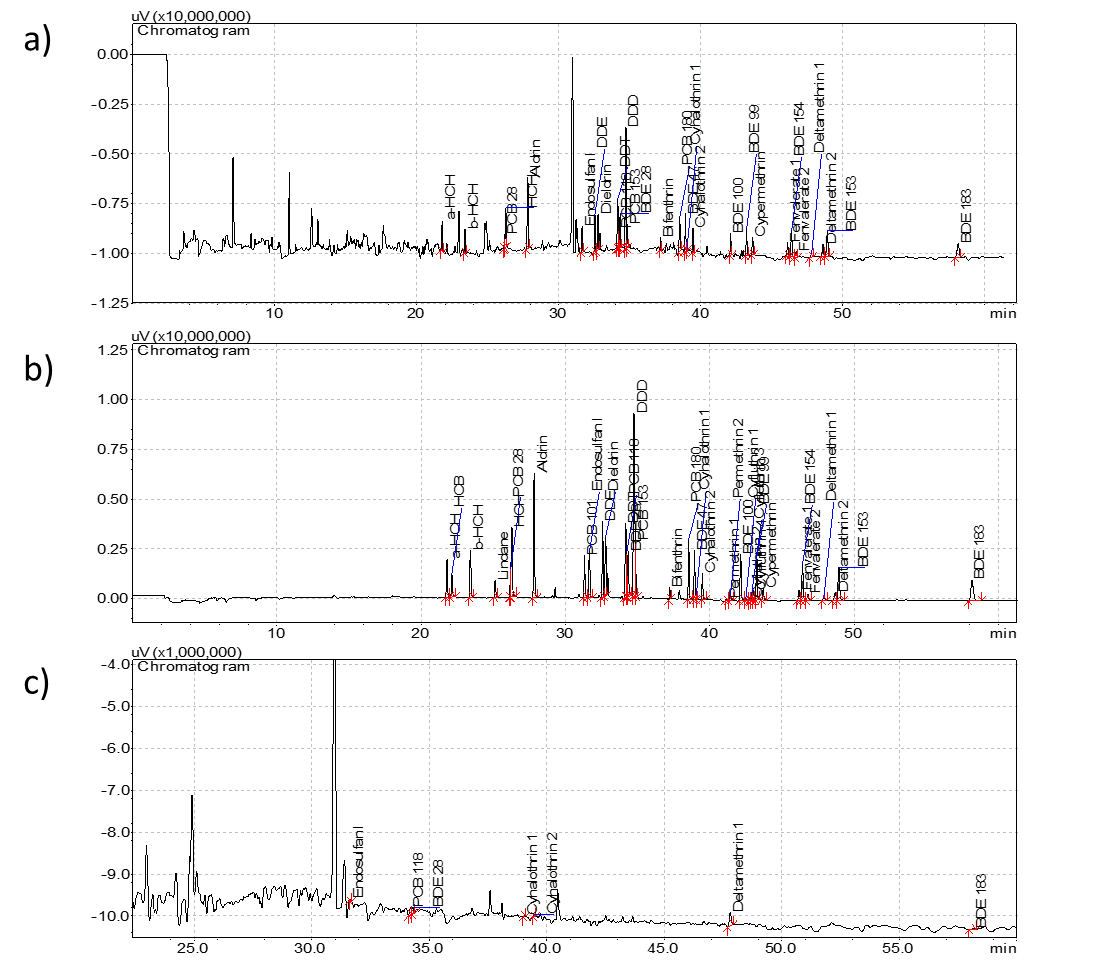


**Fig. 4S** Chromatographic data for PCBs PBDEs, OCPs, and PYRs obtained using GC-ECD. a) Chromatogram of a matrix-matched standard solution, b) chromatogram of a reagent-only standard solution, and c) chromatogram of sample A


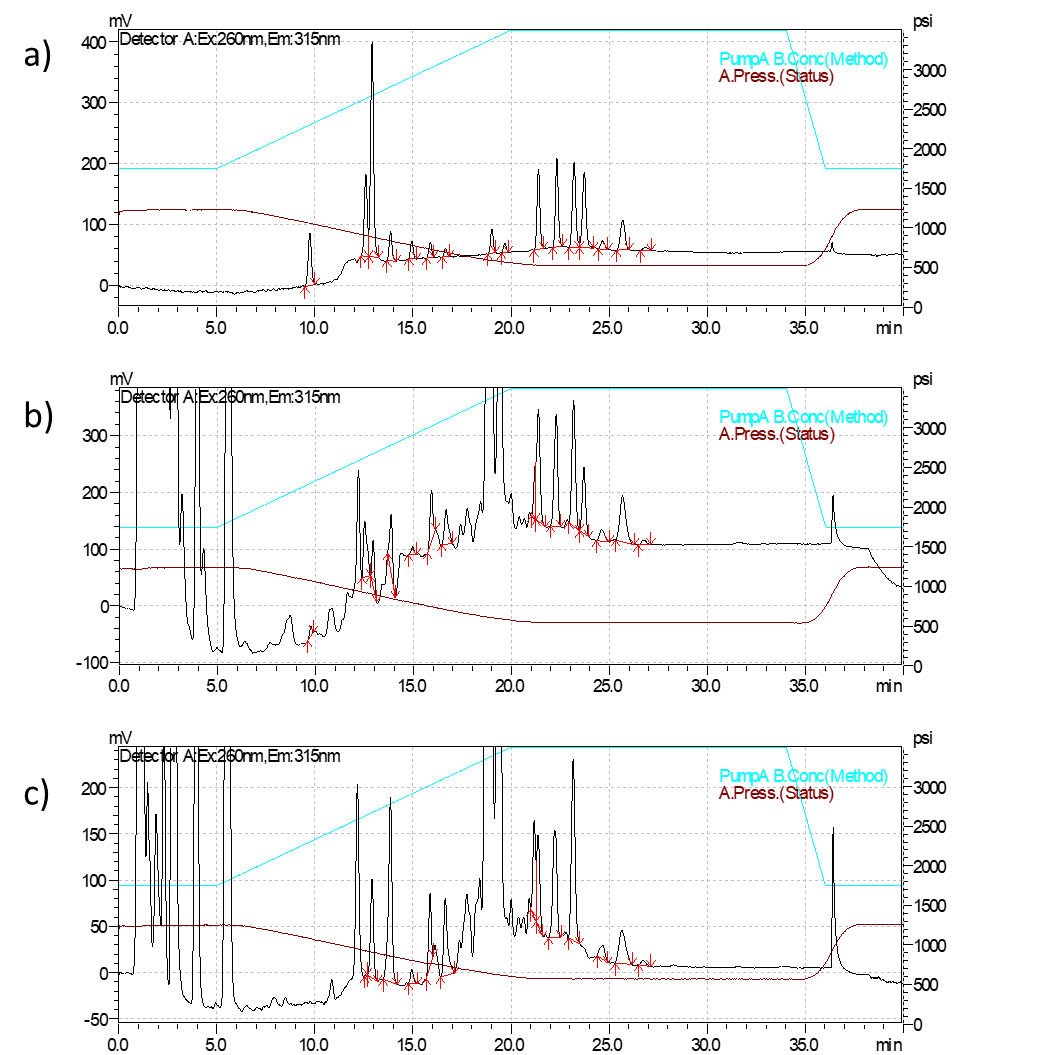


**Fig. 5S** Chromatographic data for PAHs obtained using LC-PDA-FLD. a) Chromatogram of a reagent-only standard solution, b) chromatogram of a matrix-matched standard solution, and c) chromatogram of sample C

**References**

Ademoyegun, O.T., Okoh, O.O., Okoh, A.I., 2020. Organochlorine pesticides in selected sewage sludge in South Africa: Assessment and method validation. Polish J. Environ. Stud. 29, 1021–1028. https://doi.org/10.15244/pjoes/97391

APHA, 1992. SM 3050B, in: Andrew D. Eaton, Rodger B. Baird, E.W.R. (Ed.), Standard Methods for the Examination of Water and Wastewater.

Barakat, A.O., Khairy, M.A., Mahmoud, M.R., 2017. Organochlorine pesticides and polychlorinated biphenyls in sewage sludge from Egypt. J. Environ. Sci. Heal. - Part A Toxic/Hazardous Subst. Environ. Eng. 52, 750–756. https://doi.org/10.1080/10934529.2017.1303313

CEN, 2002. EN 12457-4, in: Characterization of Waste -Leaching - Compliance Test for Leaching of Granular Waste Materias and Sludges.

Chen, C.F., Ju, Y.R., Lim, Y.C., Hsieh, S.L., Tsai, M.L., Sun, P.P., Katiyar, R., Chen, C.W., Dong, C. Di, 2019. Determination of polycyclic aromatic hydrocarbons in sludge from water and wastewater treatment plants by GC-MS. Int. J. Environ. Res. Public Health 16, 2604. https://doi.org/10.3390/ijerph16142604

Gao, L., Shi, Y., Li, W., Liu, J., Cai, Y., 2016. Occurrence and distribution of organophosphate triesters and diesters in sludge from sewage treatment plants of Beijing, China. Sci. Total Environ. 544, 143–149. https://doi.org/10.1016/j.scitotenv.2015.11.094

J.B.Jones, 2001. Laboratory Guide for conducting soil tests and plant analysis, Acta Universitatis Agriculturae et Silviculturae Mendelianae Brunensis. CRC Press LLC.

Maragou, N.C., Balayiannis, G., Karanasios, E., Markellou, E., Liapis, K., 2021. Targeted multiresidue method for the analysis of different classes of pesticides in agro-food industrial sludge by liquid chromatography tandem mass spectrometry. Molecules 26, 6888. https://doi.org/10.3390/molecules26226888

Martínez-Moral, M.P., Tena, M.T., 2014. Use of microextraction by packed sorbents following selective pressurised liquid extraction for the determination of brominated diphenyl ethers in sewage sludge by gas chromatography-mass spectrometry. J. Chromatogr. A 1364, 28–35. https://doi.org/10.1016/j.chroma.2014.08.075

Masiá, A., Vásquez, K., Campo, J., Picó, Y., 2015. Assessment of two extraction methods to determine pesticides in soils, sediments and sludges. Application to the Túria River Basin. J. Chromatogr. A 1378, 19–31. https://doi.org/10.1016/j.chroma.2014.11.079

Sánchez-Brunete, C., Miguel, E., Tadeo, J.L., 2009. Analysis of polybrominated diphenyl ethers in sewage sludge by matrix solid-phase dispersion and isotope dilution GC-MS. J. Sep. Sci. 32, 109–117. https://doi.org/10.1002/jssc.200800479

Suciu, N.A., Lamastra, L., Trevisan, M., 2015. PAHs content of sewage sludge in Europe and its use as soil fertilizer. Waste Manag. 41, 119–127. https://doi.org/10.1016/j.wasman.2015.03.018

USEPA, 1986. EPA 9045D, in: Test Methods for Evaluating Solid Waste. Springfield.

Wang, Y., Kannan, P., Halden, R.U., Kannan, K., 2019. A nationwide survey of 31 organophosphate esters in sewage sludge from the United States. Sci. Total Environ. 655, 446–453. https://doi.org/10.1016/j.scitotenv.2018.11.224
